# Structural Characterization of Bacterioferritin from *Blastochloris viridis*

**DOI:** 10.1371/journal.pone.0046992

**Published:** 2012-10-09

**Authors:** Weixiao Y. Wahlgren, Hadil Omran, David von Stetten, Antoine Royant, Sjoerd van der Post, Gergely Katona

**Affiliations:** 1 Department of Chemistry and Molecular Biology, University of Gothenburg, Göteborg, Sweden; 2 European Synchrotron Radiation Facility, Grenoble, France; 3 Institut de Biologie Structurale Jean-Pierre Ebel, CNRS CEA Université Joseph Fourier, Grenoble, France; 4 Proteomics Core Facility, University of Gothenburg, Göteborg, Sweden; National Institute for Medical Research, Medical Research Council London, United Kingdom

## Abstract

Iron storage and elimination of toxic ferrous iron are the responsibility of bacterioferritins in bacterial species. Bacterioferritins are capable of oxidizing iron using molecular oxygen and import iron ions into the large central cavity of the protein, where they are stored in a mineralized form. We isolated, crystallized bacterioferritin from the microaerophilic/anaerobic, purple non-sulfur bacterium *Blastochloris viridis* and determined its amino acid sequence and X-ray structure. The structure and sequence revealed similarity to other purple bacterial species with substantial differences in the pore regions. Static 3- and 4-fold pores do not allow the passage of iron ions even though structural dynamics may assist the iron gating. On the other hand the B-pore is open to water and larger ions in its native state. In order to study the mechanism of iron import, multiple soaking experiments were performed. Upon Fe(II) and urea treatment the ferroxidase site undergoes reorganization as seen in bacterioferritin from *Escherichia coli* and *Pseudomonas aeruginosa*. When soaking with Fe(II) only, a closely bound small molecular ligand is observed close to Fe_1_ and the coordination of Glu94 to Fe_2_ changes from bidentate to monodentate. DFT calculations indicate that the bound ligand is most likely a water or a hydroxide molecule representing a product complex. On the other hand the different soaking treatments did not modify the conformation of other pore regions.

## Introduction

**Figure 1:**
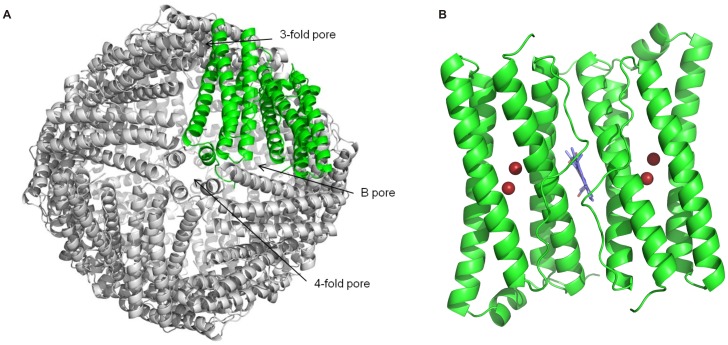
Bacterioferritin from *Blastochloris viridis*. (A) Overview of the *Bv* Bfr with 12 dimers. (B) Zoom in view of two subunits forming one homodimer. The location of the heme-b on the 2-fold axis is shown. The red spheres represent Fe ions located in the ferroxidase site of each monomer.

Bacterioferritins (Bfr) solve two important problems for bacterial cells: they reduce the concentration of toxic free Fe(II), a source of reactive oxygen species through the Fenton reaction, and provide an accessible storage of iron in a mineralized ferric form. There are many crystal structures of Bfrs available, some of which resulting from unintentional crystallization as Bfr appears to copurify frequently with other proteins. The first crystal structure of *Escherichia coli* (*Ec*) Bfr revealed that the holoprotein is made up of 24 subunits with cubic symmetry forming a hollow protein shell. [1] The mineralized ferric iron is stored within the cavity, but the catalytic conversion of ferrous iron to ferric state takes place near the ferroxidase center present in each of the 24 subunit (Figure 1). [1] The ferroxidase site is evolutionary well conserved and has binding capacity for two irons. These observations have been confirmed by the subsequent crystal structures from *Rhodobacter capsulatus* (*Rc*) [2], *Desulfovibrio desulfuricans* (*Dd*) [3], *Azotobacter vinelandii* (*Av*) [4], *Mycobacterium smegmatis* (*Ms*) [5], *Mycobacterium tuberculosis* (*Mt*) [6], *Rhodobacter sphaeroides* (*Rs*) [7] and *Pseudomonas aeruginosa* (*Pa*) [8] and revealed similar tertiary and quaternary structure. Purple bacterial bacterioferritins are especially well represented in the PDB database. [9] These versatile facultative anaerobic species can rapidly switch their metabolism from reducing photosynthetic to respiratory heterotroph mode. Ferroxidase activity provides crucial evolutionary advantage for anaerobic organisms during aerobic exposure. [10].

The exact role of the ferroxidase site is a matter of debate. It is unclear whether it participates in the iron transport directly and whether catalytic steps are coupled to this process. The minimal function of the ferroxidase site is the conversion of substrates Fe(II) and O_2_ or H_2_O_2_ to water and Fe(III). In addition for some bacterioferritins, such as *Dd* and *Pa* Bfr it has been proposed that the import of iron ions also occurs through ferroxidase site, where the conversion from Fe(II) to Fe(III) takes place. It was motivated by spectroscopic studies of iron uptake in protein solution, and by the X-ray crystallographic structural details showing that both the ‘as-isolated’ and the ‘mineralized’ structures have an empty ferroxidase center, which indicates instability. [8] On the contrary, spectroscopic and kinetic studies on *Ec* Bfr showed that the ferroxidase center is stable at all oxidation states and is required throughout the iron core formation. [11] Together with X-ray crystallographic studies, the ferroxidase center of *Ec* Bfr was suggested to function as a true catalytic cofactor, rather than as a pore for the transferring iron ion into the central cavity. [8] The iron ions were proposed to enter the cavity through other channels (see below) and only get oxidized on the internal side of the ferroxidase site. Following this idea Bfrs can be classified as possessing “loose” and “tight” ferroxidase sites where “loose” Bfrs are frequently isolated with empty ferroxidase site and bound irons are more prone to disappear upon oxidative treatments. [8] Similarly, in eukaryotic H-chain ferritins the ferroxidase center has been suggested to function as a gated site for the transfer of iron ion into the cavity after oxidation. [12].

The occupancy of the irons affects the conformation of the coordinating residues, but in addition the presence of bound ligands, the electronic state of the active site and the surrounding solvent has also a great influence. In one of the earliest mechanistic studies Macedo *et al.* found fully occupied iron sites with a bridging electron density or a water molecule appearing upon oxygen exposure and dithionite reduction respectively. [3] Crow *et al.* also observed bridging electron density between the two irons upon 65 min aerobic soaking. [11] The coordinating residues, in particular His130 can assume different rotamer conformation. In *Mt* [6] and *Av* [4] Bfr the His130 is pointing away from the ferroxidase site upon reduction. The position of the His130 side chain is not coordinating in native *Pa* Bfr, but when excess of Fe(II) was introduced in the soaking buffer His130 turned to coordinate the iron. [8] Contrarily, in the stable “as isolated” *Ec* Bfr the His130 is in coordinating position, but phosphate soaking makes His130 rotate away from the iron coordinating position. [11].

Despite of the abundant structural information available from eight unique structures from different bacterial species, questions still remain concerning the entry and exit of iron ions through the protein shell. The inner core of the *Bv* Bfr is linked to the exterior of the protein by eight 3-fold pores and six 4-fold pores. (Figure 1) The most comprehensive functional description of symmetry pores comes from studies of eukaryotic ferritins, where highly conserved negatively charged residues line across the 3-fold channels, whereas highly hydrophobic residues border the 4-fold pores. Since iron ions are attracted to negative charges it was suggested that iron enter and exit these proteins via the 3-fold pores. [13] Further supporting this theory, localized unfolding of 3-fold pores was observed in some of the crystal structures when highly conserved residues were mutated around the pores that influence pore gating. [14]–[17] When the temperature was increased or urea was added at physiological concentrations (1–10 mM), the 3-fold pores were locally unfolded, which made the iron mineral accessible to cellular reductant such as reduced flavin. [15], [18], [19].

Bacterioferritins also possess 12 heme-b molecules intercalated between the protein subunits (Figure 1B), this is the main feature that distinguishes them from eukaryotic ferritins. In contrast to the ferroxidase site, the function of the heme-b molecules is even more elusive. They were implied in the process of the releasing of iron ions from the mineralized core. [20] Very recently the presence of heme-bs was reported to increase the rate of iron core formation as well through an, as yet undefined, electron transfer mechanism. [21].

Here we report the crystal structure of bacterioferritin from *Blastochloris viridis* (*Bv* Bfr) up to 1.58 Å resolution. Since genomic information is not available from this bacterium we determined the protein sequence by a combination of redundant PCR amplification of the *Bv* Bfr gene and *de novo* peptide sequencing by mass spectrometry. To investigate the influence of solution environment on bacterioferritin different crystal soaking conditions were tested. In addition to determining the X-ray structure of the native and soaked crystals, their redox state was also monitored with UV/visible microspectrophotometry before and after X-ray exposure. The high resolution crystal structures enabled us to rationalize the electronic and protonation state of the ferroxidase site with the help of density functional theory calculations.

## Materials and Methods

### Purification and Crystallization

Crystallization of bacterioferritin from *Blastochloris viridis (B. viridis)* occurred accidentally while attempting to crystallize the photosynthetic reaction center: light harvesting 1 core complex (RC-LH1) of this organism. Cells of *B. viridis* strain ATCC 19567 were grown semianaerobically. Membranes were obtained by sonication. After removing unbroken cells by centrifugation, membranes were pelleted and resuspended in 20 mM Tris-HCl pH 8.5 to OD_1012_ = 50. Membrane proteins were extracted by addition of 2% (w/v) CHAPS and the supernatant was loaded on a DEAE column (DE52 preswollen microgranular, Whatman) pre-equilibrated with buffer A of 10 mM Tris-HCl pH 8, 5% glycerol, 0.5% CHAPS. Both *Bv* Bfr and RC-LH1 were eluted using a linear NaCl gradient from 0 to 0.5 M in buffer A. A final step of gel filtration chromatography (Sephacryl S-400, GE Healthcare) was carried out in buffer A and the colored peak containing both proteins were pooled and concentrated by Vivaspin centrifugal concentrator with a 100-kDa molecular mass cut-off.

The *Bv* Bfr crystals were formed in a reagent solution of 0.1 M HEPES pH 7.5, 0.1 M NaCl, 1.6 M (NH_4_)_2_SO_4_ using the hanging-drop vapor-diffusion method at 20°C. Red cubic-shaped crystals suitable for X-ray data collection appeared after one week. Crystal Fe-soaking experiments were performed aerobically by transferring as-isolated *Bv* Bfr crystals into freshly prepared Fe(II) soaking solution composed of the reagent solution supplemented with 50 mM FeSO_4_ and incubated for 1 hour. During the incubation period brown precipitate formed in the drops indicating the autoxidation of iron to insoluble Fe(III). Shorter incubation periods were also tested and 15 minute soaking period was found to be necessary and sufficient to develop isomorphous structural changes as observed in 1 hour incubation. (Table S2) Double-soaked crystals were prepared by aerobically soaking as-isolated *Bv* Bfr crystals in the Fe (II)-soaking solution for 1 h, followed by aerobically soaking for 15 min in a double-soaking solution of the same reagent solution containing 10 mM urea.

### X-ray Data Collection and Analysis

*Bv* Bfr crystals were flash frozen in liquid nitrogen for data collection without any cryoprotectant solution. Diffraction data were collected at 100 K with ADSC Q315r CCD detector at beamline ID29 and ID14-EH4 of the European Synchrotron Radiation Facility (ESRF). Data were indexed, integrated and scaled using XDS and XSCALE [22]. The space group F23 was chosen with unit cell dimensions a = b = c = 170.2 Å so that the electron density of heme-b could be described by one major orientation in the bacterioferritin shell. The structure was solved by molecular replacement using the program PHASER [23] of CCP4 6.1.2 Program Suite. [24] As search model the structure of PDB ID: 3GVY [25] was used. During the refinement, two iron (II) ions and one heme-b molecule were modeled to each structure. The models were then systematically improved using iterative cycles of manual rebuilding with the program Coot [26] and structure refinement with Refmac. [27] The stereochemistry of the structures was assessed with WHATCHECK [28] and PROCHECK. [29] Crystallographic data and refinement statistics are shown in Table 1. Molecular graphics was visualized by Pymol. [30].

**Table 1:** Crystallographic data and refinement statistics.

	*Bv* Bfr (as-isolated)	*Bv* Bfr (Fe-soaked)	*Bv* Bfr (double-soaked)
*Data reduction*			
Space group	F23	F23	F23
Cell constants a = b = c (Å)	170.2	170.2	170.7
Resolution (Å)^a^	19.7–1.80(1.85–1.80)	39.1–1.58(1.62–1.58)	39.2–1.68(1.72–1.68)
Total reflections	80950	2147162	2023598
Unique reflections	35784	52949	46773
Completeness (%)^a^	94.6 (98.8)	100 (100)	100 (100)
R_sym_ (%)a,b	15.5 (69.5)	7.3 (68.2)	7.4 (68.8)
<I/σ>^a^	8.9 (2.5)	28.8 (2.3)	29.7 (2.3)
R_work_ (%)^c^	15.5	15.6	16.5
R_free_ (%)^c^	19.2	17.4	18.6
Average *B*-factor (Å^2^)			
Main-chain	16.3	17.0	18.4
Side-chain	21.9	21.6	23.1
Heme	22.4	23.5	23.9
Solvent	32.8	31.7	32.1
All atoms	20.8	20.7	22.0
3-fold pore^d^	17.7	18.2	19.3
4-fold pore^e^	16.1	17.6	18.4
Occupancy (%)			
Fe_1_	100	100	100
Fe_2_	40	100	70
R.m.s. deviation fromideal bond length (Å)	0.029	0.029	0.030
R.m.s. deviation fromideal bond angles (°)	2.29	3.30	2.15
Ramachandran plot(% by PROCHECK)			
Most favored	97.9	97.2	97.9
Additionally allowed	2.1	2.8	2.1
Generously allowed	0	0	0
Disallowed	0	0	0
PDB reference code	4am2	4am5	4am4

a Values in parentheses indicate statistics for the highest resolution shell.

b *R*_sym_ = ∑ |Io−<I>|/∑ Io×100%, where Io is the observed intensity of a reflection and <I> is the average intensity obtained from multiple observations of symmetry related reflections.

c *R*_factor = _∑ ||F_obs_|-|F_calc_||/∑ |F_obs_|×100%.

d Average *B*-factor of amino acids shown in Figure 6B.

e Average *B*-factor of amino acids shown in Figure 7B.

### UV/visible Absorption Microspectrophotometry of Bv Bfr Crystals

Crystal UV/visible absorption spectra were recorded at the ID29S Cryobench laboratory [31] before and after X-ray exposure at beamline ID29 (ESRF). Approximately 50 µm sized crystals were selected for spectroscopic analysis and Fe(II) soaking was performed as described above. Crystals were dipped for 2–3 s in cryoprotected soaking solution (or mother liquor for native crystals) containing 20% glycerol before flash cooling. All spectra were recorded on cryocooled crystals at 100 K.

### Sequencing

The following primers were used in the degenerate PCR experiments: forward primer (5′ATGAAGGGCGACTCGAAGGTSATCGARTAYCTS 3′) and reverse complementery primer (5′ YTCSAGGAAGTCGATGTGCTTYTCYTC 3′), where S = C or G, R = A or G, Y = T or C.

### Quantum Chemical Modeling of the Fe(II) Soaked Bv Bfr Ferroxidase Center

DFT calculations were performed using the program PC GAMESS/Firefly QC package [32], which is partially based on the GAMESS (US) [33] source code. The B3LYP functional was employed with the 6–31G(d,p) basis set for the open-shell unrestricted Hartree-Fock model optimization.

The simulated active site is illustrated in Figure S1, and it contains simplified analogs of Glu18, Glu51, Glu94, Glu127, His54, His130, Wat2, Fe_1_, Fe_2_ and the tested ligand bound to Fe_1_. Initial atomic coordinates are based on the Fe(II) soaked state. The calculation was performed in vacuum and in order to simulate the constraints of the protein scaffold, the Cartesian coordinates of atoms marked red were fixed during the optimization. The optimization was tested using different ligands bound to Fe_1_: both a diatomic oxygen molecule and a single oxygen atom were modeled in the electron density followed by refinement in Refmac5 [34]. Hydrogen atoms were incorporated using the program Avogadro [35]. Initial few iterations of the optimization was performed with the basis set MINI and initial orbitals were generated by the Hückel method. The preoptimized coordinates were further optimized with the 6–31G(d,p) basis set until convergence. The optimized geometry was analyzed with the program Molden [36].

## Results and Discussion

**Figure 2:**
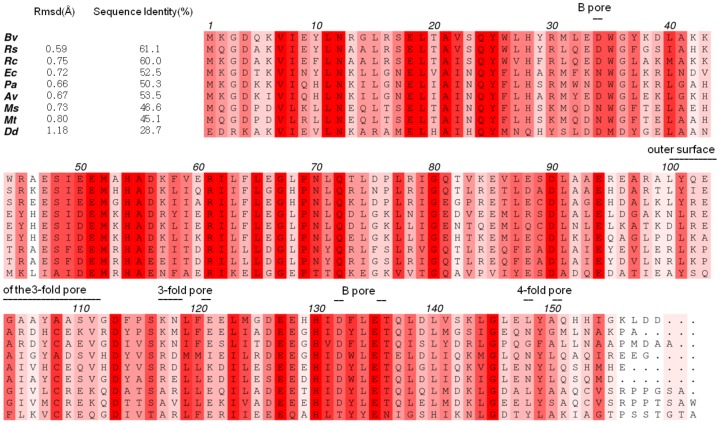
Multiple sequence alignment. Multiple sequence alignment of bacterioferritin from Blastochloris viridis (Bv), Rhodobacter sphaeroides (Rs), Rhodobacter capsulatus (Rc), Escherichia coli (Ec), Pseudomonas aeruginosa (Pa), Azotobacter vinelandii (Av), Mycobacterium smegmatis (Ms), Mycobacterium tuberculosis (Mt) and Desulfovibrio desulfuricans (Dd). The more conserved positions are colored darker red. The figure was produced with the program Aline. [41].

Bacterioferritin from *Blastochloris viridis* was isolated from its native host and crystallized. Native crystals *Bv* Bfr diffracted to 1.8 Å resolution. The structure was solved by molecular replacement using the Bfr model from *Rhodobacter sphaeroides* (PDB id: 3GVY). The final X-ray diffraction data and refinement statistics of the native “as isolated” dataset are summarized in Table 1. In the absence of genomic information an initial guess of the amino acid sequence was derived directly from the electron density. Based on this preliminary estimate one pair of redundant PCR primers were constructed based on the well defined conserved regions of the sequence. After successful amplification, the translated sequence of the PCR product covered 68.5% of the protein sequence. The remaining regions of the N- and C-terminus were resolved by *de novo* sequencing using tandem mass spectrometry after enzymatic digestion with either trypsin or chymotrypsin to generate overlapping peptides, confirming the complete sequence of *Bv* Bfr (Table S1). Where there was an uncertainty due to equal amino acid residue masses (leucine and isoleucine), the real-space electron density correlation of the side chains was used to break the ambiguity (Ile8, Ile131 and Ile154). By the combination of electron density analysis, nucleotide sequencing and tandem mass spectrometry the complete amino acid sequence of *Bv* Bfr was recovered (Figure 2). Its closest sequence and structural homologue is *Rs* Bfr with an amino acid sequence identity of 61.1%. The structural similarity of *Bv* Bfr to *Rs* Bfr (PDB id: 3GVY) is evidenced by the low, 0.59 Å root mean square deviation between structurally equivalent C_α_ positions after least square superposition (using the native ‘as isolated’ crystal structure in Table 1). Most difference in amino acid sequence and structural alterations are located in the loop regions, in particular at the positions located close the 3- and 4-fold symmetry axes of the protein shell.

### Ferroxidase Site

**Figure 3:**
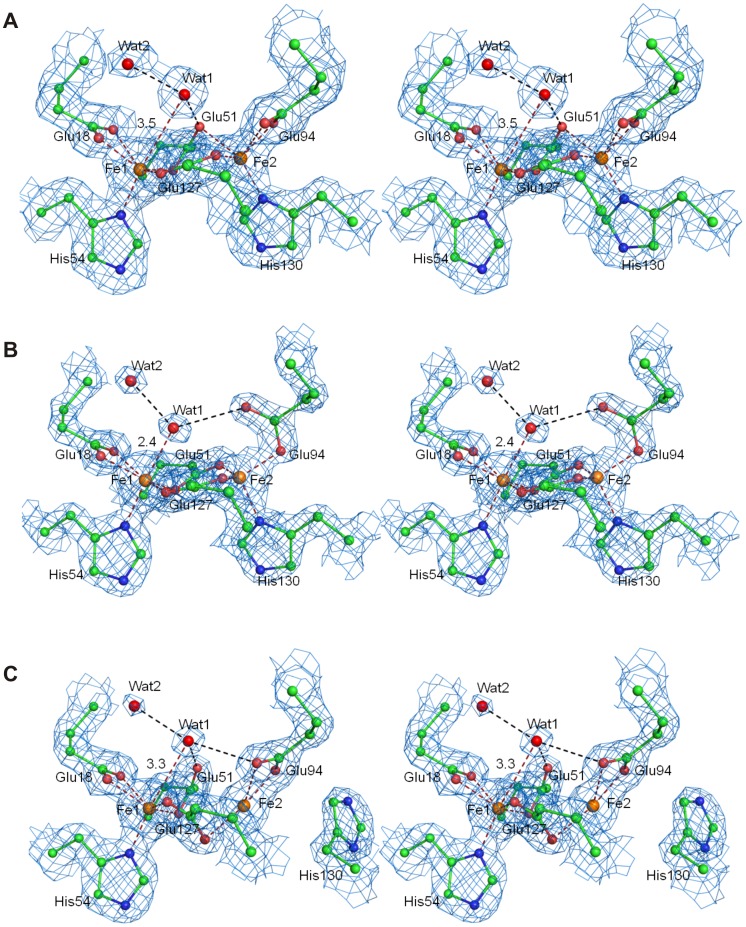
The ferroxidase center of *Bv* Bfr in stereo view of electron density in 2mF_o_-DF_c_ maps. (A) The structure of as-isolated, (B) Fe-soaked and (C) double-soaked *Bv* Bfr active site, respectively. The 2mF_o_-DF_c_ map (blue) is contoured at 1σ, 2σ and 1.5σ, respectively. The dashed red lines show the coordinating bonds to the iron atoms and the dashed black lines indicate hydrogen bonds.

In *Bv* Bfr the ferroxidase active site consists of highly conserved amino acids: His54 and Glu18 are terminal ligands to iron 1 (Fe_1_), His130 and Glu94 are terminal ligands to iron 2 (Fe_2_), and Glu127 and Glu51 are bridging ligands (Figure 3). Fe_1_ has direct access to the outside of the protein shell with ordered water molecules in the vicinity. Fe_2_ on the other hand is located closer to the core of the bacterioferritin. In the “as-isolated” structure the ferroxidase center is fully occupied at the Fe_1_ site and 40% occupied at Fe_2_.

Native crystals of *Bv* Bfr were also subjected to Fe(II) and urea containing soaking environment before flash-cooling in liquid nitrogen (see full description in Materials and Methods section). Although originally we planed to study the effect of soaking on the pores of the Bfr shell (metal binding and local unfolding), the different treatments primarily affected the content and conformation of the ferroxidase center, while the rest of the bacterioferritin structure remained intact. A fully occupied ferroxidase center was present in the “Fe(II)-soaked” structure, while the Fe_2_ site had slightly higher occupancy of 60±10% in three “double-soaked” crystals compared to “as-isolated” crystals. (Table 1 and Table S2).

A secondary iron binding site near residues His46 and Asp50 was not observed in contrast to *Ec* bacterioferritin. [11] In the compared species in Figure 2, histidine is a common, but not exclusive residue at position 46 and position 50 is occupied by either glutamate or aspartate depending on the species of origin. In *Bv* Bfr Ala46 and Glu50 are unlikely to form a binding site suggesting that the secondary iron binding site may be specific to only a subset of Bfrs. We have not found unusually strong (>1.5 e^−/^Å^3^ in final 2mF_o_-DF_c_ maps) and highly coordinated (≥3 ligands within 2.7 Å) solvent electron density peaks using Coot [27] in the three *Bv* Bfr states, which might correspond to an alternative iron binding site.

**Figure 4:**
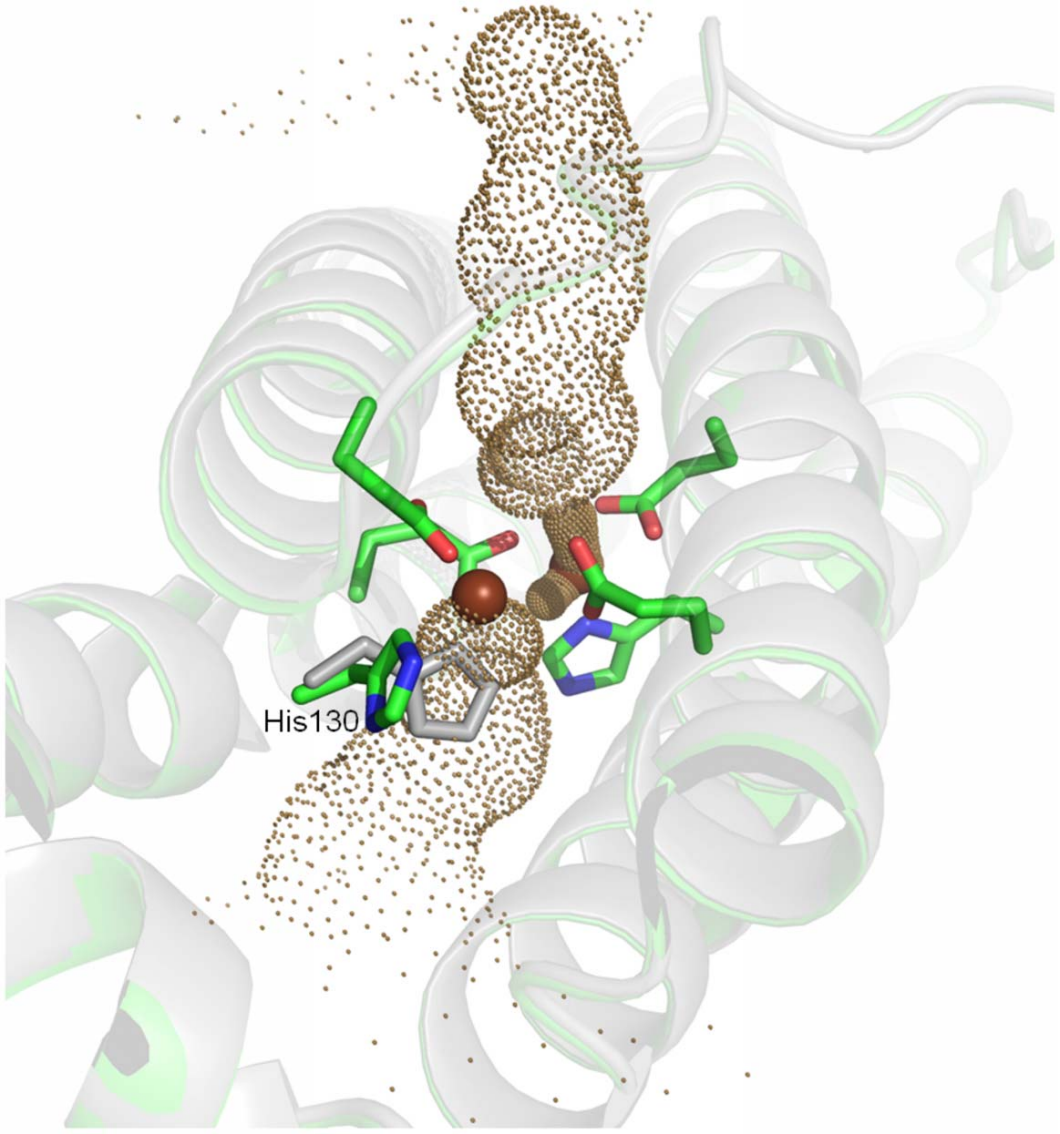
Ferroxidase site in the open and closed state. His130 from ‘as-isolated’ structure (*gray*) is rotated toward the ferroxidase center to facilitate binding of Fe_2_, while in the double-soaked structure (*green*) the His130 is in its non-coordinative conformation. Hole2 channel through the ferroxidase site was generated using the double-soaked structure with both Fe_1_ and Fe_2_ removed.

In *Bv* Bfr, the Fe_1_ binding sites were fully occupied in all three structures, while Fe_2_ site displayed variability depending on the soaking environment. This is not a general behavior in all bacterioferritins, for example in *Dd* Bfr iron at Fe_1_ position is depleted upon aerobic oxidation [3], while Fe_2_ site is fully occupied indicating that both iron sites may have limited stability. The soaking conditions also affected the coordinating residues. Most notably His130 rotated away from the Fe_2_ site upon “double-soaking” treatment in three out of three treated crystals. Non-ligated conformation of His130 was not observed in any of the “as isolated” and “Fe(II)-soaked” crystal structures. The conformational change opens access to the inner side of Bfr as illustrated on Figure 4 using the Hole2 representation. [37] In other Bfrs, except that of *Ec*, the bound irons do not appear to function as a permanent cofactor. In *Pa* [8], *Ms* [6] and *Bv* Bfr the Fe_2_ site can become accessible from inside of the core because its coordinating histidine (His130) can assume two different conformations. The changes in iron occupancy appear to be connected to reorganization of coordinating residues.

The iron-iron distance is 3.97 Å for the native, “as isolated” protein, 3.81 Å in the Fe-soaked and 3.92 Å in the double soaked structure. A long iron-iron distance such as that observed in native and double soaked *Bv* Bfr, has been reported when the irons are reduced and not bridged by any electron density. [3], [11] On the other hand bridging molecules significantly reduce the iron-iron distance by 0.2–0.3 Å. [3], [11] In the Fe(II) soaked *Bv* Bfr the distance is shorter, but not to the same extent as irons with bridging ligands (3.63 Å [11] and 3.71 Å [3]). The iron-iron distance may be shorter because Glu94 coordinating Fe_2_ changes from bidentate coordination to monodentate and the free oxygen atom of the carboxyl group makes a hydrogen bond to the ligand of Fe_1_. This additional weak hydrogen bond that derives from the unique monodentate conformation of Glu94 may substitute the strong bridging of ligands observed in previous work [3], [11] and exert a weaker force that move the iron atoms together.

**Figure 5:**
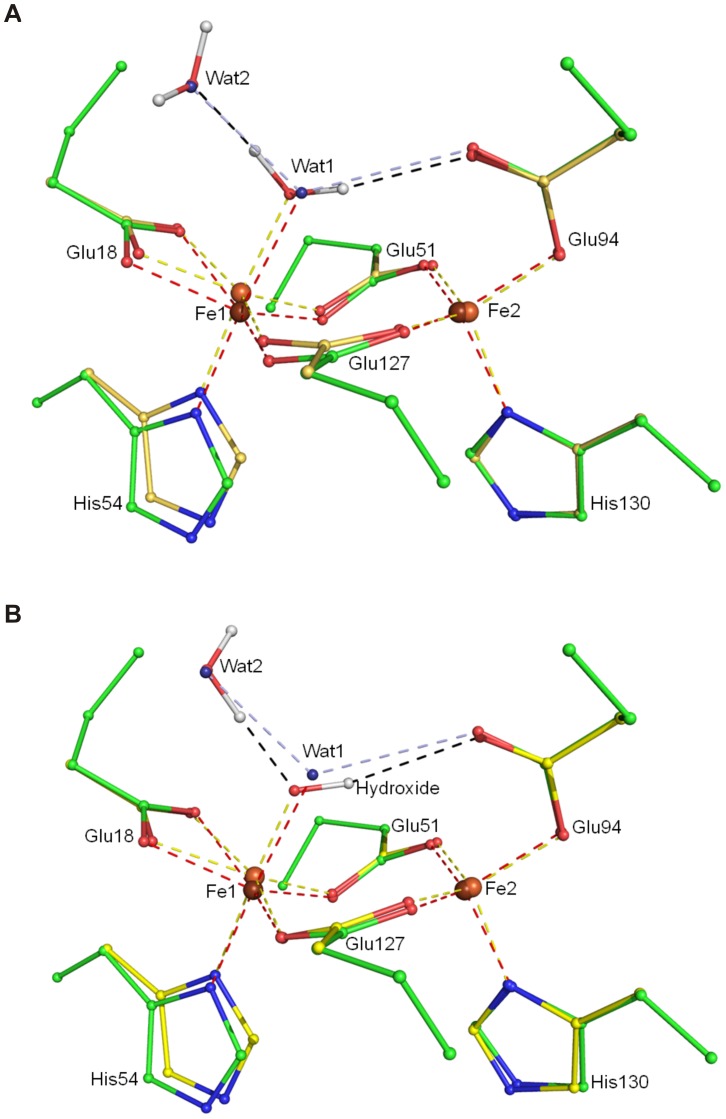
DFT calculations. Stick-ball representation of ferroxidase center from DFT calculations superimposed on the Fe-soaked crystal structure. The ligand to Fe_1_ is (A) a water molecule, with mixed valence and high multiplicity; (B) a hydroxide ion, with Fe_1_ reduced and Fe_2_ oxidized and both at high spin. The model with *green* and *yellow* carbon atoms represents the crystal structure and the DFT optimized coordinates, respectively.

Knowing the precise redox state of the protein in each crystal form is of prime importance in a structural analysis. To this end, we performed UV/visible absorption microspectrophotometry on ‘as isolated’ and Fe(II) soaked crystals. We expected our aerobically isolated crystals to be in a fully oxidized state with an empty ferroxidase active site, but absorption spectra showed that the hemes-b are in a predominantly reduced state as judged from the sharp peak at 558 nm, although a shoulder at 564 nm indicates that a fraction of hemes are oxidized (Figure S2A, black curve). The spectroscopic signature after X-ray data collection is unchanged (Figure S2A, red curve), hence our structure of ‘as isolated’ Bfr contains mostly hemes in the reduced state. In contrast, the Fe(II) soaked crystals are completely oxidized before X-ray exposure (Figure S2B, black curve) as illustrated by the broad peak at 564 nm. However, the hemes are quickly reduced with an exposure to the X-ray beam as short as 1 s (Figure S2B, red curve). Consequently, our structure of Fe(II) soaked Bfr is counter-intuitively in the same redox state as that of ‘as isolated’ Bfr. Indeed, the heme-b molecules do not change conformation or position and their crystallographic B-factor does not change significantly upon different soaking conditions even when they are normalized against the average B-factor of all atoms (Table 1 and S2). In brief, the electronic state of crystal structures from different soaking treatments may be more similar than one would predict from the UV/visible absorption spectrum before X-ray exposure.

**Figure 6:**
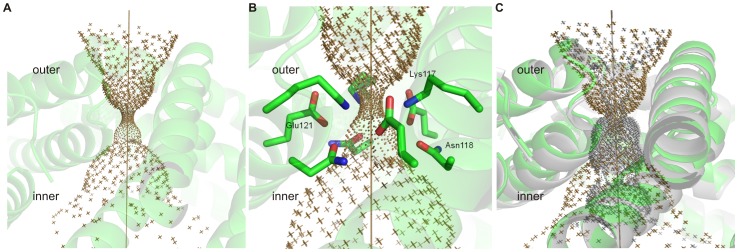
Hole2 representations of the 3 fold pore. (A) Overview of the *Bv* Bfr 3-fold pore (B) constraining residues in the *Bv* Bfr 3-fold pore (C) Superposition of *Bv* Bfr (*brown*) and *Ec* Bfr 3-fold pore (*grey*) (PDB entry 2Y3Q) [42].

Our soaking experiments recovered two distinct states of the ferroxidase active site. A ligand density is present near Fe_1_ forming either a tight or a weak interaction with it depending on the trapping condition. Bridging electron density was observed in *Ec* Bfr after long aerobic soak of anaerobicaly prepared crystals. [11] Crow *et al*. tentatively assigned this electron density as µ-oxo or µ-hydroxo bridge analogously to the one observed in ribonucleotide reductase [38]. In the multiple NCS copies of the active site of *Ec* Bfr, the distance of the bridging moiety is between 3.5–2.5 Å to Fe_1_ and 3.0–2.6 Å to Fe_2_. [11] In the “as isolated” and “double soaked” *Bv* Bfr the ligand-Fe_1_ distance is located within this range, while the ligand-Fe_2_ distance is longer, making the ligand interaction with Fe_1_ and Fe_2_ slightly weaker and more symmetric (Table 2). Contrarily, in the Fe(II) soaked *Bv* Bfr the ligand-Fe_1_ distance is considerably shorter at only 2.4 Å whereas the ligand-Fe_2_ distance remains relatively long at 3.3 Å.

**Table 2:** Interatomic distances in the different *Bv* Bfr structures compared to the DFT optimized distances from Compounds A and B from Figure S1.

Interatomicdistances (Å)	As-isolated	Fe(II)soaked	Double-soaked	DFT calculation	
				H_2_O	OH^−^
Fe_1_-Fe_2_	3.96	3.81	3.92	3.98	3.93
Fe_1_-O_ε1_ Glu18	2.47	2.23	2.30	2.20	2.28
Fe_1_-O_ε2_ Glu18	2.11	2.16	2.17	1.94	1.97
Fe_1_-O_ε2_ Glu51	2.14	2.07	2.05	1.91	1.96
Fe_1_-O_ε2_ Glu127	2.05	2.11	1.96	2.09	2.16
Fe_1_-N_δ1_ His54	1.97	2.04	2.03	1.96	2.03
Fe_1_-O Wat1	3.48	2.39	3.32	1.97	1.78
Fe_2_-O_ε1_ Glu94	2.75	2.86	2.83	2.76	2.71
Fe_2_-O_ε2_ Glu94	1.88	1.99	1.97	1.86	1.88
Fe_2_-O_ε1_ Glu51	2.57	1.98	2.94	1.97	1.97
Fe_2_-O_ε1_ Glu127	2.08	1.83	2.71	1.92	1.92
Fe_2_-N_δ1_ His130	1.88	1.99	−	1.93	1.93
Fe_2_-O Wat1	3.41	3.33	3.55	3.64	3.55
Wat1-O_ε1_ Glu51	2.30	3.03	2.38	3.16	3.13
Wat1-O_ε1_ Glu94	3.65	3.06	3.08	3.32	3.48

A further difference between the native structure and the Fe(II) soaked state that Glu51 rotates to an iron bridging position from a perpendicular orientation which is more suitable for hydrogen bonding to Wat1. In the double soaked structure Glu127 also rotates towards Fe_1_, which leaves only Glu94 to strongly coordinate Fe_2_. As a partial compensation the hydroxyl group of Tyr25 moves closer to the Fe_2_ position. The apparent correlation of Glu51 and Wat1 position and reduced occupancy at the Fe_2_ site may indicate a mechanism assisting the release of Fe_2_: as the water product shifts to a more distal position and Glu51 switches from coordinating Fe_2_ to hydrogen bonding Wat1, the Fe_2_ coordinating sphere weakens leading to iron release.

**Figure 7:**
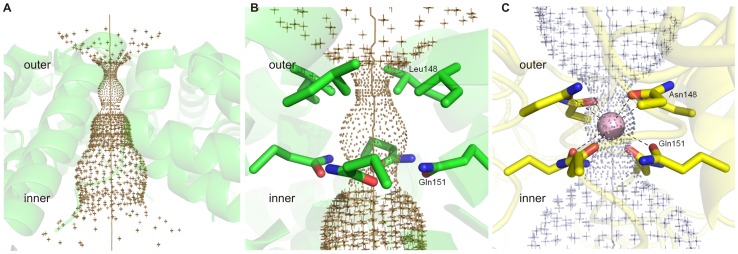
Hole2 representations of the 4 fold pore. (A) Overview of the *Bv* Bfr 4-fold pore (B) Zoom onto the residues immediately surrounding the 4-fold pore in *Bv* Bfr and (C) *Pa* Bfr 4-fold pore with a potassium ion (*pink*) modeled in the center of the pore (PDB entry 3ISF) [8].

**Figure 8:**
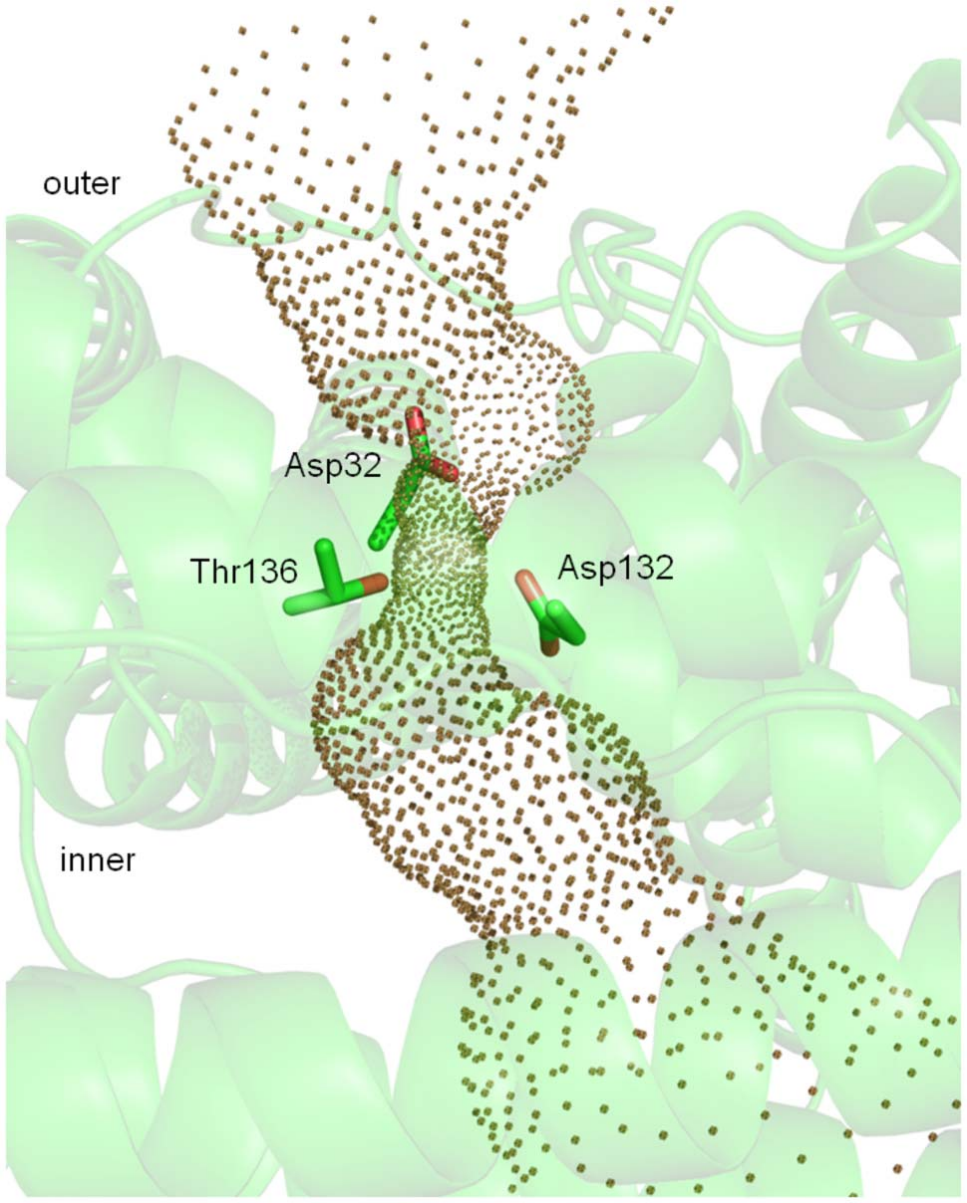
Hole2 representation of the *Bv* Bfr B-pore. The side chains of amino acid residues surrounding the constricted region of the pore are also indicated.

In the Fe(II) soaked structure Fe_1_ is octahedrally coordinated, while Fe_2_ is surrounded by near perfect tetrahedral coordination, which also means Glu94 switches from bidentate to an unusual perpendicular monodentate binding. In order to rationalize the nature of the closely bound ligand as well as the electronic and protonation states of the active site, a search was carried out using a set of DFT calculations on the active site removed from the Fe(II) soaked structure. In place of Wat1 a number of candidate atoms were introduced (corresponding to water, hydroxide ion, dioxygen and a hydroperoxo moiety). Of all calculations (Text S1, Text S2, Tables S3 and S4) two configurations yielded plausible agreement with the experimental coordinates. Wat2 at a fixed position (Figure 3B) was also included in the calculations as the presence of solvent molecules were shown to be important for the accurate simulation of the electronic and protonation state of ligands bound to iron. [39] In the first calculation represented in Figure S1A a water molecule was placed at the position of Wat1. The monodentate position of Glu94 and the tetrahedral coordination around Fe_2_ was remarkably well maintained when the diiron cluster had a mixed valence at the highest multiplicity of 10. The root mean square deviation of free atomic position between theory and experiment is 0.25 Å, close to the experimental error. (Figure 5A) On the other hand the Fe_1_-O distance is optimized to be substantially shorter (1.97 Å) in contrast to the experimental distance of 2.39 Å (Table 2). When a hydroxide ion was placed at Wat1 position (Figure S1B) Fe_1_-O distance became even shorter (1.78 Å), than the experimental distance. Nevertheless, the best agreement with the experimental data (rmsd 0.20 Å) was obtained with the hydroxide ligand, when the diiron center had a mixed valence and high multiplicity (10) similarly to the electronic state of the best model with the water ligand. (Figure 5B) This geometric optimization also maintained the monodentate coordination of Glu94 to Fe_2_.

The diiron site in the Fe(II) soaked configuration clearly favors high spin state, the lower spin states deviated more from experimental geometry and the total energy of the optimized system was always higher. Of the larger ligands tested, oxygen was not able to bind to oxidized and mixed valence active sites. In the reduced state the coordination to Fe_1_ was maintained, but no bridge formation was observed and the distal oxygen pointed in a direction that did not agree with the shape of the electron density. In summary the Fe(II) soaked crystal structure, UV/visible absorption spectrum and DFT simulations support the presence of a water or hydroxide ligand coordinated primarily with Fe_1_ in a mixed valence active site with little Δ splitting between the d orbitals.

### Conformation and Amino Acid Composition of the Three- and Four-fold Pores

The roles of 3- and 4-fold channels in the bacterioferritins are not obvious. In particular, the residues lining across these pores are not highly conserved. The 3-fold channels in the bacterioferritins are surrounded by both positively and negatively charged residues. *Bv* Bfr appears to be an exception as the outer rim of the 3-fold pore (region 110–120) contains only one negatively charged residue and most residues in this loop are hydrophobic. Even in the closest homolog *Rs* Bfr three positive and three negative residues are located in the corresponding region. In other Bfrs listed in Figure 2 the number of charged residues ranges from 3 to 6.

In eukaryotic ferritins however the rate of Fe^2+^ exit increases coincidentally with the localized unfolding of 3-fold pores when a chelator was presented on the outside of the frog ferritin H and human ferritin H. [18], [19] The double soaking protocol did not drive similar local reorganization in the 3-fold pore regions in *Bv* Bfr crystals as in eukaryotic ferritins (Figure S3). Crystallographic B-factors on the other hand are locally higher upon both Fe(II) and double soaked treatments at the 3-fold and 4-fold pores, but the significance of the change could only be shown for the residues forming the 4-fold pore (Table 3).

**Table 3:** Normalized crystallographic B-factors of the pore forming residues upon “Fe-soaked” and “double soaked” treatments.

	“As isolated”	“Fe-soaked”	“double soaked”
<B_3-fold_>/<B_total_>	0.83±0.02	0.90±0.03	0.89±0.10
<B_4-fold_>/<B_total_>	0.77±0.01	0.86±0.01	0.86±0.04
P value of Welch’s t-test [43]	between “Fe-soaked” and “as isolated” data	between “double soaked” and “as isolated” data	between “Fe-soaked” and “double soaked” data
<B_3-fold_>/<B_total_>	0.10	0.36	0.88
<B_4-fold_>/<B_total_>	0.05	0.05	0.90

In contrast to the subtle B-factor changes in the pore forming residues double soaking treatment profoundly changed the conformation of the ferroxidase site compared to the Fe(II) soaked state as discussed previously. In all three states of *Bv* Bfr, electron density consistent with the presence of ions was not found in any of the 3- and 4-fold pores, indicating that these pores are empty. Figure 6 and 7 show the shape of the channels through the proposed pores visualized by the program Hole2. [40] Using this representation in *Bv* Bfr the 3- and 4-fold pores have an hourglass shape from outside of protein shell into to the center cavity, though the 4-fold pore has a shorter outer part and longer inner part, while the two sides of 3-fold pore are approximately symmetric (see Figure 6A and 8A). From the outside the 3-fold pore starts with a diameter at approximately 3.5 Å. The narrow internal pore is in the middle of the protein shell, and is capped by side chains of three Lys117 residues constricting the channel to less than 1.2 Å. After the constriction point the internal pore becomes wider and is lined by the side chains of three Glu121 and the constrained region ends with three Asn118 residues (Figure 6B). When compared to *Ec* Bfr the 3-fold pore is only constrained at one point and peripheral residues block the pore less (Figure 6C).

For the 4-fold pore, the entrance of the narrow part positioned closer to the external surface of the protein shell, and is capped by side chains of four Leu148 residues, with a diameter less than 1.2 Å, and followed by four Gln151 residues which widen the pore to a diameter approximately 1.8 Å. Immediately after the layer of glutamines, the pore becomes broad to a diameter larger than 2.5 Å, enough for a water molecule to occupy (Figure 7A and 7B). In *Bv* Bfr electron density was not observed in the 4-fold pore, but different cations were modeled in the 4-fold pores in *Pa* Bfr and *Av* Bfr, coordinated by eight oxygen atoms from the side chains of four Asn148 and four Gln151 residues (Figure 7C). [4], [8].

### B pore

Although the 3-fold and 4-fold pores are attractive candidates for the entry and exit of iron and phosphate ions the surrounding residues are not very well conserved in sequence. On the other hand the B pore or B site previously identified in *Dd* Bfr [3] is more preserved in the course of evolution. This pore is accessible in all three *Bv* Bfr states (Figure 8). Due to the sequence conservation negative charged residues are concentrated around the B pore. The narrowest part of the pore has a diameter of 1.4 Å, and is bordered by Asp34 from one of the monomers, Asp132 and Thr136 from an adjacent monomer. (Figure 8) Well-ordered water molecules connected by hydrogen network were observed in the channel; however electron density does not indicate any iron ion inside the pore. The only metal ions (octahedral Mg^2+^ ions) ligated by water molecules were observed in the B pore of *Av* Bfr structure. [4] Nevertheless the B pore is a likely, well conserved candidate for efficient transport of iron and phosphate ions.

### Conclusion

While we cannot fully exclude the role of 3-fold/4-fold pores in iron gating the observed pore conformations do not easily allow the passage of iron ions even though structural dynamics may assist the iron transport. This notion is supported by the significant local increase of B-factors in the 4-fold forming residues upon Fe(II) and double soaking treatment. With that said, more plausible iron entry points are the ferroxidase site and the B-pore. The B-pore is wide enough for iron to pass though in its native conformation and conformational change is not required for this. The ferroxidase active site is blocked for irons in the native state, but becomes instable upon “double soaking” treatment. Since the iron occupancy can vary at the active site and iron ions have wide enough channels for binding from both side of the protein shell when assisted by the conformational change of His130. Moreover, the ferroxidase active site revealed a closely bound ligand to Fe_1_ and an unusual monodentate coordination of Glu94 to Fe_2_ upon Fe(II) soaking. We identified the ligand as a water molecule or hydroxide ion, revealing a potential product complex resulting from the ferroxidase reaction.

## Supporting Information

Figure S1 **Schematic representation of the compounds used in the DFT simulations.** Cartesian coordinates of atoms marked with *red* were fixed during optimization.
(TIF)

Figure S2 **UV/visible spectrum of** ***Bv*** **Bfr crystals.** (A) UV/visible spectrum of native as isolated *Bv* Bfr crystals before (*black*) and after (*red*) 100 s X-ray exposure at beamline ID29, ESRF (5% transmission, X-ray flux: 9.6×10^10^ ph/s). (B) Crystal spectrum of Fe(II) soaked *Bv* Bfr before (*black*) and after (*red*) 1 s X-ray exposure at beamline ID29, ESRF (5% transmission, X-ray flux: 1.2×10^11^ ph/s).
(TIF)

Figure S3 **Superposition of the pore forming residues in the 3-fold and 4-fold pore.** (A) 3-fold pore (B) 4-fold pore. The native ‘as isolated’ structure, the Fe(II) soaked and double soaked structures are marked with *green*, *blue* and *yellow* respectively.
(TIF)

Table S1 **Identified peptides of protease digested** ***Bv*** **Bfr fragments.**
(DOC)

Table S2 **Data and refinement statistics of the additional crystal structures provided in the Supporting information.**
(DOC)

Table S3 **Total energy, charge and spin distributions derived from the DFT calculations.**
(DOC)

Table S4 **Structural similarity to the crystallographic positions and key distances in the DFT optimized geometry.**
(DOC)

Dataset S1 **Coordinate and structure factor files of the additional crystal structure named in Table S2 as Dataset S1.**
(ZIP)

Dataset S2 **Coordinate and structure factor files of the additional crystal structure named in Table S2 as Dataset S2.**
(ZIP)

Dataset S3 **Coordinate and structure factor files of the additional crystal structure named in Table S2 as Dataset S3.**
(ZIP)

Dataset S4 **Coordinate and structure factor files of the additional crystal structure named in Table S2 as Dataset S4.**
(ZIP)

Dataset S5 **Coordinate and structure factor files of the additional crystal structure named in Table S2 as Dataset S5.**
(ZIP)

Text S1 **Method description of** ***de novo*** **sequencing by tandem mass spectrometry and DFT calculations.**
(DOC)

Text S2 **Cartesian coordinates derived from the DFT calculations listed in Table S3.**
(DOC)

## References

[1] Frolow F, Kalb AJ, Yariv J (1994) Structure of a unique twofold symmetric haem-binding site. Nat Struct Biol 1: 453–460. 7664064 10.1038/nsb0794-453

[2] Cobessi D, Huang LS, Ban M, Pon NG, Daldal F, et al (2002) The 2.6 A resolution structure of Rhodobacter capsulatus bacterioferritin with metal-free dinuclear site and heme iron in a crystallographic ‘special position’. Acta Crystallogr D Biol Crystallogr 58: 29–38. 11752777 10.1107/s0907444901017267 PMC4615704

[3] Macedo S, Romao CV, Mitchell E, Matias PM, Liu MY, et al (2003) The nature of the di-iron site in the bacterioferritin from Desulfovibrio desulfuricans. Nature Structural Biology 10: 285–290. 12627224 10.1038/nsb909

[4] Swartz L, Kuchinskas M, Li H, Poulos TL, Lanzilotta WN (2006) Redox-dependent structural changes in the Azotobacter vinelandii bacterioferritin: new insights into the ferroxidase and iron transport mechanism. Biochemistry 45: 4421–4428. 16584178 10.1021/bi060146w

[5] Janowski R, Auerbach-Nevo T, Weiss MS (2008) Bacterioferritin from Mycobacterium smegmatis contains zinc in its di-nuclear site. Protein Sci 17: 1138–1150. 18445621 10.1110/ps.034819.108 PMC2442004

[6] Gupta V, Gupta RK, Khare G, Salunke DM, Tyagi AK (2009) Crystal structure of Bfr A from Mycobacterium tuberculosis: incorporation of selenomethionine results in cleavage and demetallation of haem. PLoS One 4: e8028. 19946376 10.1371/journal.pone.0008028 PMC2777505

[7] Dominy BN, Singh MK (2010) Thermodynamic resolution: How do errors in modeled protein structures affect binding affinity predictions? Proteins-Structure Function and Bioinformatics 78: 1613–1617. 10.1002/prot.22691 20201067

[8] Weeratunga SK, Lovell S, Yao H, Battaile KP, Fischer CJ, et al (2010) Structural studies of bacterioferritin B from Pseudomonas aeruginosa suggest a gating mechanism for iron uptake via the ferroxidase center. Biochemistry 49: 1160–1175. 20067302 10.1021/bi9015204 PMC2852880

[9] Berman HM, Westbrook J, Feng Z, Gilliland G, Bhat TN, et al (2000) The Protein Data Bank. Nucleic Acids Res 28: 235–242. 10592235 10.1093/nar/28.1.235 PMC102472

[10] Romao CV, Regalla M, Xavier AV, Teixeira M, Liu MY, et al (2000) A bacterioferritin from the strict anaerobe Desulfovibrio desulfuricans ATCC 27774. Biochemistry 39: 6841–6849. 10841764 10.1021/bi992525d

[11] Crow A, Lawson TL, Lewin A, Moore GR, Le Brun NE (2009) Structural basis for iron mineralization by bacterioferritin. J Am Chem Soc 131: 6808–6813. 19391621 10.1021/ja8093444

[12] Levi S, Luzzago A, Cesareni G, Cozzi A, Franceschinelli F, et al (1988) Mechanism of ferritin iron uptake: activity of the H-chain and deletion mapping of the ferro-oxidase site. A study of iron uptake and ferro-oxidase activity of human liver, recombinant H-chain ferritins, and of two H-chain deletion mutants. J Biol Chem 263: 18086–18092. 3192527

[13] Hempstead PD, Yewdall SJ, Fernie AR, Lawson DM, Artymiuk PJ, et al (1997) Comparison of the three-dimensional structures of recombinant human H and horse L ferritins at high resolution. J Mol Biol 268: 424–448. 9159481 10.1006/jmbi.1997.0970

[14] Treffry A, Bauminger ER, Hechel D, Hodson NW, Nowik I, et al (1993) Defining the roles of the threefold channels in iron uptake, iron oxidation and iron-core formation in ferritin: a study aided by site-directed mutagenesis. Biochem J 296 (Pt 3): 721–728. 10.1042/bj2960721 PMC1137755 7506527

[15] Theil EC, Liu XS, Tosha T (2008) Gated Pores in the Ferritin Protein Nanocage. Inorganica Chim Acta 361: 868–874. 19262678 10.1016/j.ica.2007.08.025 PMC2350241

[16] Hasan MR, Tosha T, Theil EC (2008) Ferritin contains less iron (59Fe) in cells when the protein pores are unfolded by mutation. Journal of Biological Chemistry 283: 31394–31400. 18805796 10.1074/jbc.M806025200 PMC2581568

[17] Takagi H, Shi D, Ha Y, Allewell NM, Theil EC (1998) Localized unfolding at the junction of three ferritin subunits. A mechanism for iron release? Journal of Biological Chemistry 273: 18685–18688. 9668036 10.1074/jbc.273.30.18685

[18] Liu XS, Patterson LD, Miller MJ, Theil EC (2007) Peptides selected for the protein nanocage pores change the rate of iron recovery from the ferritin mineral. Journal of Biological Chemistry 282: 31821–31825. 17785467 10.1074/jbc.C700153200

[19] Liu X, Jin W, Theil EC (2003) Opening protein pores with chaotropes enhances Fe reduction and chelation of Fe from the ferritin biomineral. Proc Natl Acad Sci U S A 100: 3653–3658. 12634425 10.1073/pnas.0636928100 PMC152977

[20] Yasmin S, Andrews SC, Moore GR, Le Brun NE (2011) A new role for heme, facilitating release of iron from the bacterioferritin iron biomineral. J Biol Chem 286: 3473–3483. 21106523 10.1074/jbc.M110.175034 PMC3030353

[21] Wong SG, Abdulqadir R, Le Brun NE, Moore GR, Mauk AG (2012) Fe-haem bound to Escherichia coli bacterioferritin accelerates iron core formation by an electron transfer mechanism. Biochem J 444: 553–560. 22458666 10.1042/BJ20112200

[22] Kabsch W (1993) Automatic Processing of Rotation Diffraction Data from Crystals of Initially Unknown Symmetry and Cell Constants. Journal of Applied Crystallography 26: 795–800.

[23] McCoy AJ, Grosse-Kunstleve RW, Storoni LC, Read RJ (2005) Likelihood-enhanced fast translation functions. Acta Crystallogr D Biol Crystallogr 61: 458–464. 15805601 10.1107/S0907444905001617

[24] Bailey S (1994) The CCP4 Suite - Programs for Protein Crystallography. Acta Crystallographica Section D-Biological Crystallography 50: 760–763. 10.1107/S0907444994003112 15299374

[25] Nam KH, Xu Y, Piao S, Priyadarshi A, Lee EH, et al (2010) Crystal structure of bacterioferritin from Rhodobacter sphaeroides. Biochem Biophys Res Commun 391: 990–994. 19968959 10.1016/j.bbrc.2009.12.003

[26] Murshudov GN, Vagin AA, Dodson EJ (1997) Refinement of macromolecular structures by the maximum-likelihood method. Acta Crystallographica Section D-Biological Crystallography 53: 240–255. 10.1107/S0907444996012255 15299926

[27] Emsley P, Cowtan K (2004) Coot: model-building tools for molecular graphics. Acta Crystallographica Section D-Biological Crystallography 60: 2126–2132. 10.1107/S0907444904019158 15572765

[28] Hooft RWW, Vriend G, Sander C, Abola EE (1996) Errors in protein structures. Nature 381: 272–272. 8692262 10.1038/381272a0

[29] Laskowski RA, MacArthur MW, Moss DS, Thornton JM (1993) PROCHECK: a program to check the stereochemical quality of protein structures. Journal of Applied Crystallography 26: 283–291.

[30] DeLano W The PyMOL Molecular Graphics System, Version 1.3. Schrödinger, LLC.

[31] Royant A, Carpentier P, Ohana J, McGeehan J, Paetzold B, et al (2007) Advances in spectroscopic methods for biological crystals. 1. Fluorescence lifetime measurements. Journal of Applied Crystallography 40: 1105–1112.

[32] Granovsky AA Firefly version 7.1.G. Available: http://classic.chem.msu.su/gran/firefly/index.html.Accessed 2012 Sep 18.

[33] Schmidt MW, Baldridge KK, Boatz JA, Elbert ST, Gordon MS, et al (1993) General Atomic and Molecular Electronic-Structure System. Journal of Computational Chemistry 14: 1347–1363.

[34] Murshudov GN, Skubak P, Lebedev AA, Pannu NS, Steiner RA, et al (2011) REFMAC5 for the refinement of macromolecular crystal structures. Acta Crystallographica Section D-Biological Crystallography 67: 355–367. 10.1107/S0907444911001314 PMC3069751 21460454

[35] Hanwell MD, Curtis DE, Lonie DC, Vandermeersch T, Zurek E, et al (2012) Avogadro: An advanced semantic chemical editor, visualization, and analysis platform. J Cheminform 4: 17. 22889332 10.1186/1758-2946-4-17 PMC3542060

[36] Schaftenaar G, Noordik JH (2000) Molden: a pre- and post-processing program for molecular and electronic structures. J Comput Aided Mol Des 14: 123–134. 10721501 10.1023/a:1008193805436

[37] Smart OS, Breed J, Smith GR, Sansom MS (1997) A novel method for structure-based prediction of ion channel conductance properties. Biophys J 72: 1109–1126. 9138559 10.1016/S0006-3495(97)78760-5 PMC1184496

[38] Ling J, Sahlin M, Sjoberg BM, Loehr TM, Sanders-Loehr J (1994) Dioxygen is the source of the mu-oxo bridge in iron ribonucleotide reductase. J Biol Chem 269: 5595–5601. 8119895

[39] Katona G, Carpentier P, Niviere V, Amara P, Adam V, et al (2007) Raman-assisted crystallography reveals end-on peroxide intermediates in a nonheme iron enzyme. Science 316: 449–453. 17446401 10.1126/science.1138885

[40] Smart OS, Goodfellow JM, Wallace BA (1993) The pore dimensions of gramicidin A. Biophys J. 65: 2455–2460. 10.1016/S0006-3495(93)81293-1 PMC1225986 7508762

[41] Bond CS, Schuttelkopf AW (2009) ALINE: a WYSIWYG protein-sequence alignment editor for publication-quality alignments. Acta Crystallogr D Biol Crystallogr 65: 510–512. 19390156 10.1107/S0907444909007835

[42] Antonyuk SV, Hough MA (2011) Monitoring and validating active site redox states in protein crystals. Biochim Biophys Acta 1814: 778–784. 21215826 10.1016/j.bbapap.2010.12.017

[43] Welch BL (1947) The Generalization of ‘Student’s’ Problem When Several Different Population Varlances Are Involved. Biometrika 34: 28–35. 20287819 10.1093/biomet/34.1-2.28

