# Supplementary figures and images for: Structural Characterization of Bacterioferritin from Blastochloris viridis
Source: PLoS One. 2012 Oct 9;7(10):e46992. doi: 10.1371/journal.pone.0046992 (PMC3467274; doi:10.1371/journal.pone.0046992)

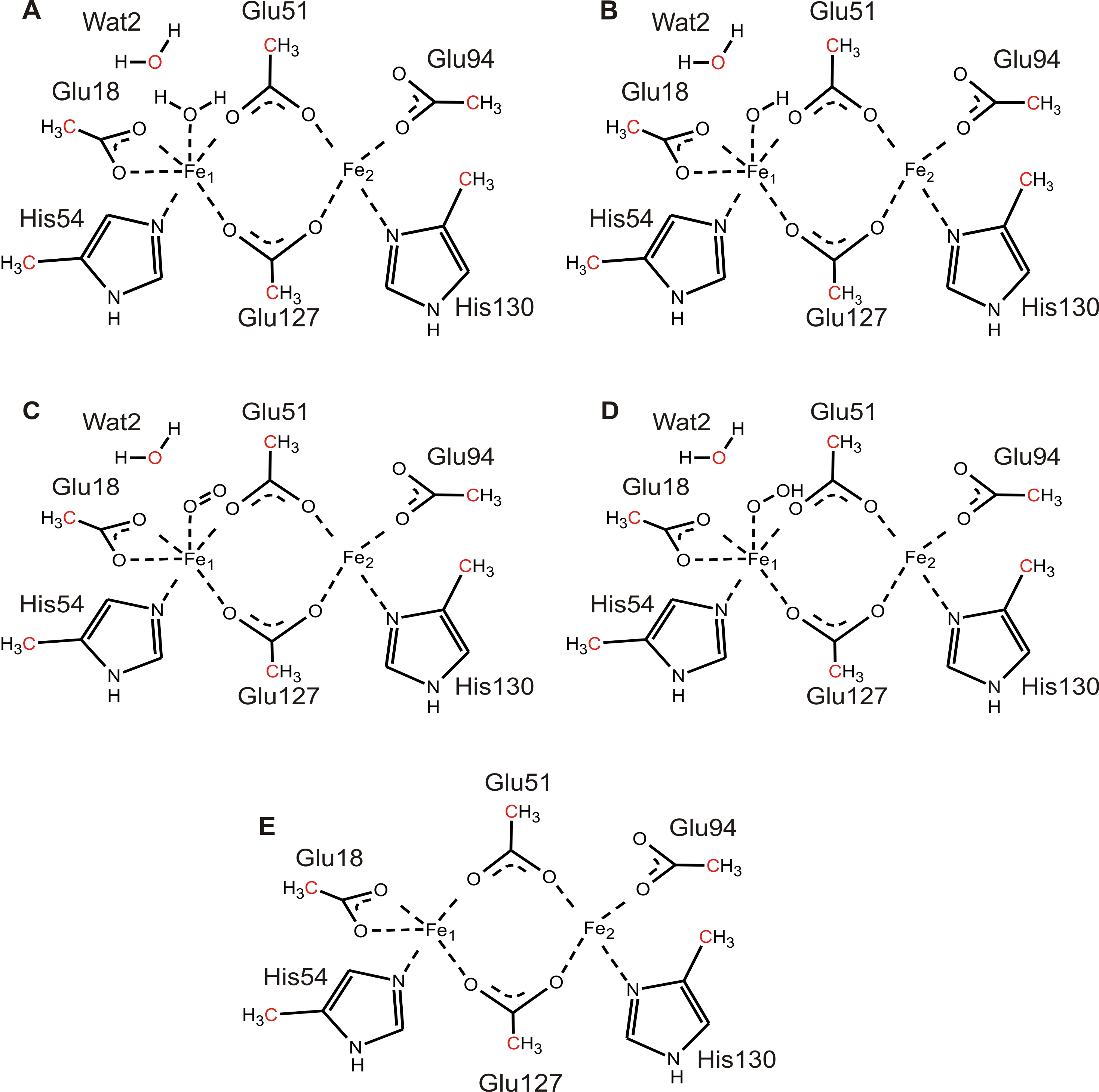

Supplement: Figure S1 — Schematic representation of the compounds used in the DFT simulations. Cartesian coordinates of atoms marked with red were fixed during optimization. (TIF) [file pone.0046992.s001.tif]

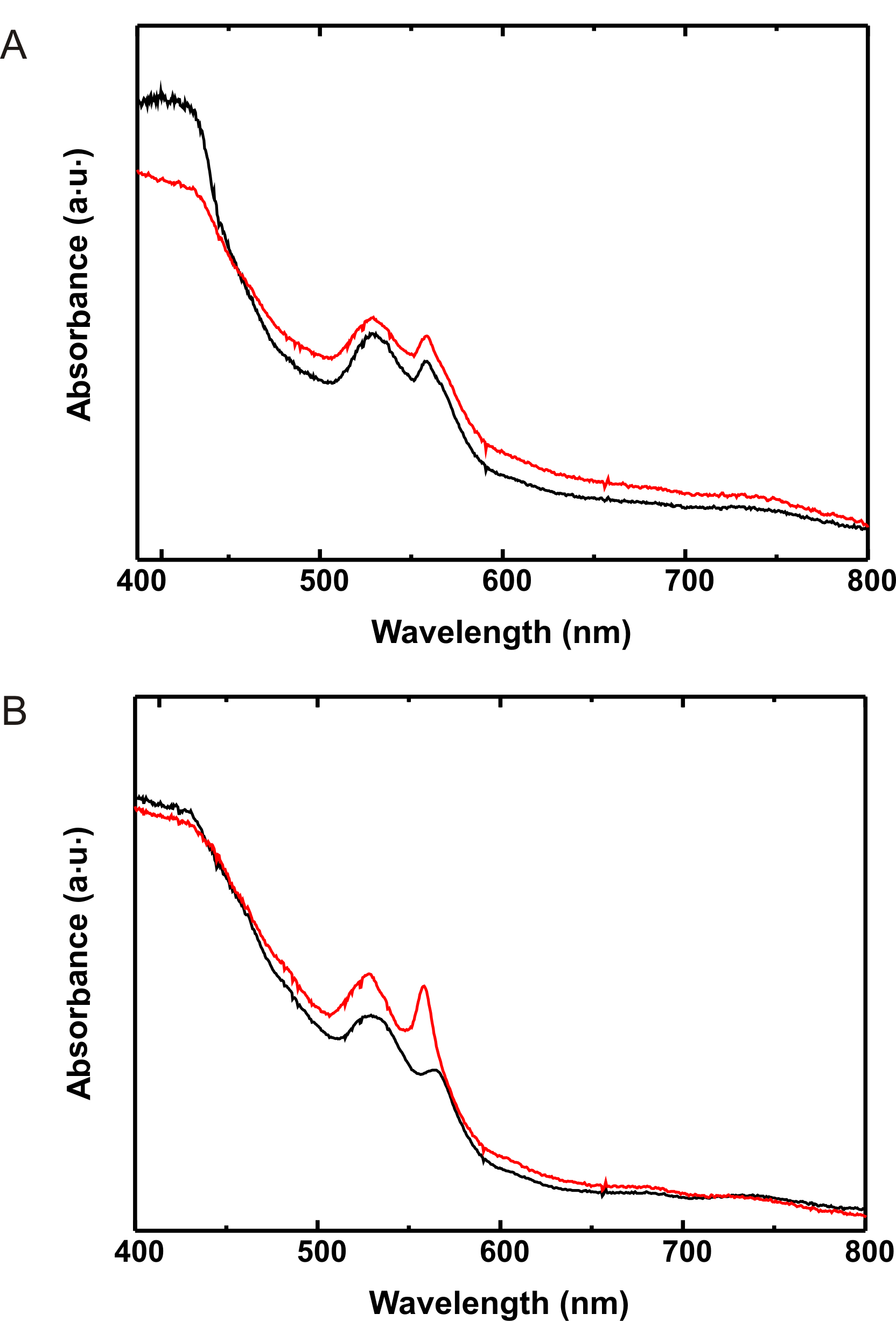

Supplement: Figure S2 — UV/visible spectrum of Bv Bfr crystals. (A) UV/visible spectrum of native as isolated Bv Bfr crystals before (black) and after (red) 100 s X-ray exposure at beamline ID29, ESRF (5% transmission, X-ray flux: 9.6×10^10^ ph/s). (B) Crystal spectrum of Fe(II) soaked Bv Bfr before (black) and after (red) 1 s X-ray exposure at beamline ID29, ESRF (5% transmission, X-ray flux: 1.2×10^11^ ph/s). (TIF) [file pone.0046992.s002.tif]

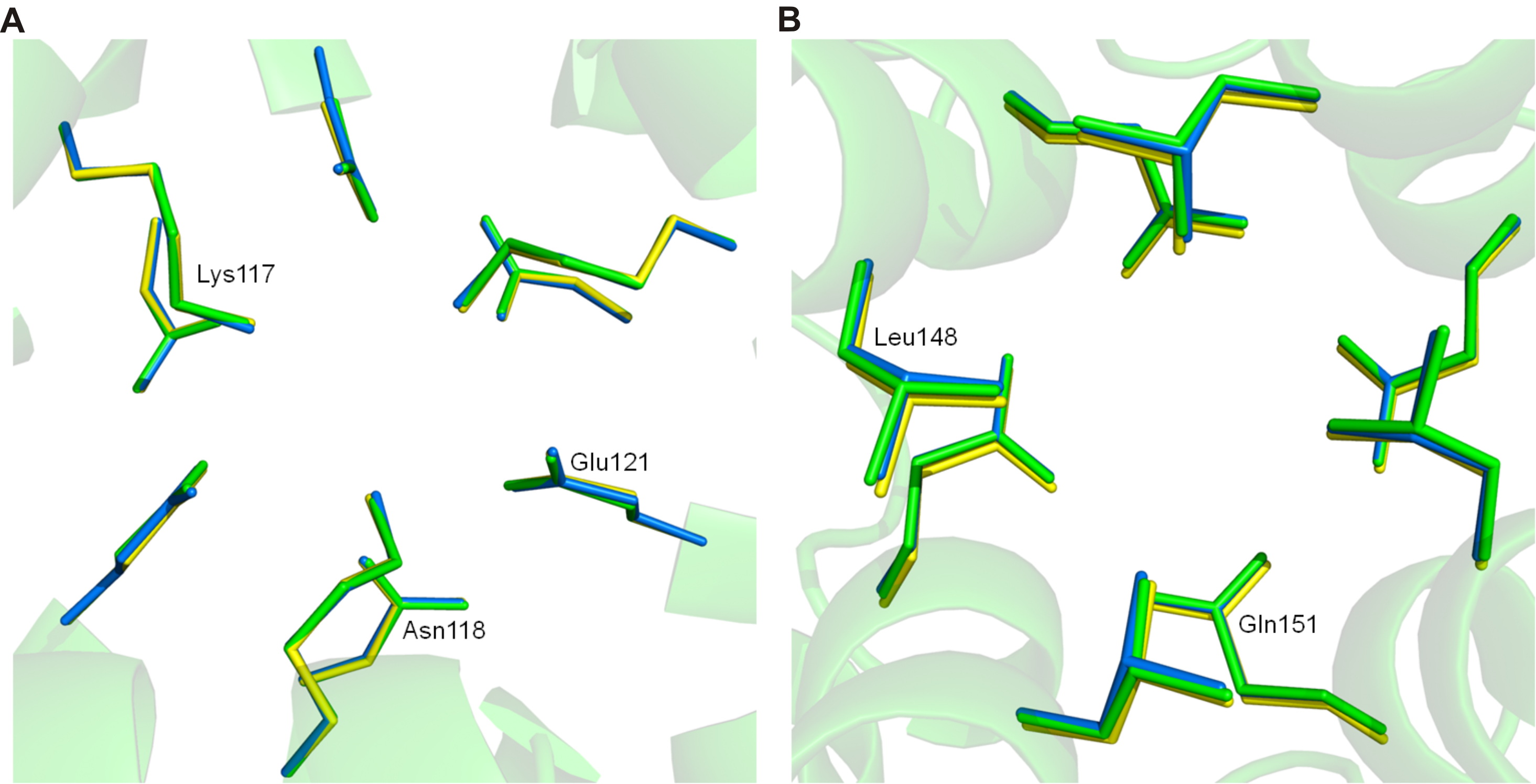

Supplement: Figure S3 — Superposition of the pore forming residues in the 3-fold and 4-fold pore. (A) 3-fold pore (B) 4-fold pore. The native ‘as isolated’ structure, the Fe(II) soaked and double soaked structures are marked with green, blue and yellow respectively. (TIF) [file pone.0046992.s003.tif]
