# Supplementary material for: Structural Characterization of Bacterioferritin from Blastochloris viridis
Source: PLoS One. 2012 Oct 9;7(10):e46992. doi: 10.1371/journal.pone.0046992 (PMC3467274; doi:10.1371/journal.pone.0046992)
Supplement: Table S1 — Identified peptides of protease digested Bv Bfr fragments. (DOC) [file pone.0046992.s004.doc]

**Table S1** Identified peptides of protease digested *Bv* Bfr fragments.

| Start-  End | Sequence | MW observed^a^ | MW expected | ion score | Enzyme^b^ |
| --- | --- | --- | --- | --- | --- |
| 1-13 | -.MKGDQKVIEYLNR | 1592.8344 | 1592.8354 | 70 | T |
| 1-20 | -.MKGDQKVIEYLNRGLRSELT | 2365.2435 | 2365.2423 | 53 | CT |
| 1-24 | -.MKGDQKVIEYLNRGLRSELTAVSQ | 2750.4394 | 2750.4385 | 61 | CT |
| 17-30 | SELTAVSQYWLHYR | 1751.8631 | 1751.8651 | 93 | T |
| 31-42 | MLEDWGYKDLAK | 1483.7017 | 1483.7034 | 79 | T |
| 31-43 | MLEDWGYKDLAKK | 1595.8017 | 1595.8033 | 60 | T |
| 44-61 | WRAESIEEMAHADKFVER | 2203.0503 | 2203.048 | 93 | T |
| 45-58 | RAESIEEMAHADKF | 1632.7566 | 1632.7569 | 79 | T |
| 46-61 | AESIEEMAHADKFVER | 1860.8676 | 1860.8695 | 113 | T |
| 62-78 | ILFLEGLPNLQTLDPLR | 1951.1169 | 1951.1142 | 83 | T |
| 78-91 | RIGQTVKEVLESDL | 1585.8679 | 1585.8675 | 65 | CT |
| 79-95 | IGQTVKEVLESDLAAER | 1856.9805 | 1856.9843 | 136 | T |
| 79-98 | IGQTVKEVLESDLAAEREAR | 2213.1683 | 2213.1651 | 95 | T |
| 85-95 | EVLESDLAAER | 1230.6098 | 1230.6092 | 78 | T |
| 85-98 | EVLESDLAAEREAR | 1586.792 | 1586.79 | 112 | T |
| 99-117 | ALYQEGAAYAASVGDFPSK | 1943.9292 | 1943.9265 | 121 | T |
| 108-120 | AASVGDFPSKNLF | 1351.6776 | 1351.6772 | 56 | CT |
| 110-123 | SVGDFPSKNLFEEL | 1580.7731 | 1580.7722 | 59 | CT |
| 121-138 | EELMGDEEHHLDFLETQL | 2183.9688 | 2183.9681 | 71 | CT |
| 134-144 | LETQLDLVSKL | 1257.7183 | 1257.718 | 56 | CT |
| 143-159 | LGLQLYAQHHLGKLDD.- | 1819.9512 | 1819.9575 | 73 | T |
| 149-159 | YAQHHLGKLDD.- | 1295.6268 | 1295.6258 | 89 | CT |

^a^ Masses are in Dalton

^b^ Protease used for digestion: trypsin (T) or chymotrypsin (CT)
