# Supplementary material for: Structural Characterization of Bacterioferritin from Blastochloris viridis
Source: PLoS One. 2012 Oct 9;7(10):e46992. doi: 10.1371/journal.pone.0046992 (PMC3467274; doi:10.1371/journal.pone.0046992)
Supplement: Table S2 — Data and refinement statistics of the additional crystal structures provided in the Supporting information. (DOC) [file pone.0046992.s005.doc]

**Table S2** Data and refinement statistics of the additional crystal structures provided in the Supporting information.

|  | Bv Bfr  (as-isolated) | | Bv Bfr  (Fe-soaked)^a^ | Bv Bfr  (double-soaked) | |
| --- | --- | --- | --- | --- | --- |
| Data reduction |  |  |  |  |  |
| Space group | F23 | F23 | F23 | F23 | F23 |
| Cell constants  a=b=c(Å) | 171.75 | 171.28 | 170.83 | 173.4 | 173.24 |
| Resolution (Å)^b^ | 42.9-2.50  (2.57-2.50) | 42.8-2.35  (2.41-2.35) | 51.5-2.25  (2.38-2.25) | 39.8-1.55  (1.59-1.55) | 35.4-2.30  (2.44-2.30) |
|  |  |  |  |  |  |
| Total reflections | 139176 | 129885 | 389851 | 2306433 | 625881 |
| Unique reflections | 14646 | 17445 | 19680 | 61428 | 18744 |
| Completeness(%)^b^ | 100 (100) | 100 (100) | 100 (100) | 98.5(99.7) | 97.5(97.5) |
| R_sym_ (%)^b,c^ | 13.6(64.5) | 15.6(63.4) | 11.5(58.0) | 7.0(66.4) | 13.2(54.8) |
| <I / σ>^b^ | 18.1(4.0) | 13.8(3.3) | 24.0(2.7) | 31.9(2.5) | 28.2(12.4) |
| R_work_ (%)^d^ | 15.8 | 15.4 | 16.3 | 16.8 | 20.4 |
| R_free_ (%)^d^ | 21.1 | 20.1 | 21.3 | 19.4 | 25.2 |
| Average *B*-factor (Å)  Main-chain  Side-chain  Heme  Solvent  All atoms  3-fold pore^e^  4-fold pore^f^ |  |  |  |  |  |
|  | 23.8 | 19.8 | 26.4 | 17.1 | 27.5 |
|  | 27.1 | 23.2 | 30.5 | 21.9 | 33.2 |
|  | 31.3 | 26.6 | 33.5 | 23.9 | 33.2 |
|  | 31.2 | 30.5 | 31.8 | 42.3 | 36.8 |
|  | 25.6 | 22.2 | 29.0 | 22.0 | 27.4 |
|  | 20.8  19.6 | 18.1  16.8 | 26.8  25.2 | 17.7  18.3 | 27.0  24.6 |
| Occupancy (%)  Fe_1_  Fe_2_ |  |  |  |  |  |
|  | 100  40 | 100  40 | 100  100 | 100  50 | 100  60 |
| R.m.s. deviation from  Ideal bond length (Å) | 0.016 | 0.018 | 0.018 | 0.029 | 0.019 |
| R.m.s. deviation from  Ideal bond angles (°) | 2.34 | 2.39 | 2.39 | 3.33 | 2.40 |
| Ramachandran plot  (% by PROCHECK) |  |  |  |  |  |
| Most favored | 97.9 | 97.9 | 97.9 | 97.9 | 97.9 |
| Additionally allowed | 2.1 | 2.1 | 2.1 | 2.1 | 2.1 |
| Generously allowed | 0 | 0 | 0 | 0 | 0 |
| Disallowed | 0 | 0 | 0 | 0 | 0 |
| **SI** | **Dataset-S1** | **Dataset-S2** | **Dataset-S3** | **Dataset-S4** | **Dataset-S5** |

^a^ The crystal was incubated in Fe(II)-soaking solution for 15 minutes.

^b^ Values in parentheses indicate statistics for the highest resolution shell.

^c^ R_sym_= ∑ |I_o_ - ‹I›|/∑ I_o_ x 100%, where I_o_ is the observed intensity of a reflection and ‹I› is the average. intensity obtained from multiple observations of symmetry related reflections.

^d^  R_factor_ = ∑ ||F_obs_|-|F_calc_||/∑ |F_obs_| x 100 %.

^e^ Average *B*-factor of amino acids shown in Figure 6B.

^f^ Average *B*-factor of amino acids shown in Figure 7B.
