# Supplementary material for: Structural Characterization of Bacterioferritin from Blastochloris viridis
Source: PLoS One. 2012 Oct 9;7(10):e46992. doi: 10.1371/journal.pone.0046992 (PMC3467274; doi:10.1371/journal.pone.0046992)
Supplement: Table S3 — Total energy, charge and spin distributions derived from the DFT calculations. (DOC) [file pone.0046992.s006.doc]

**Table S3** Total energy, charge and spin distributions derived from the DFT calculations.

| Modeled compound | Charge | Multiplicity | Total energy (Ha) | Mulliken charge population | | | | Mulliken spin population | | | |
| --- | --- | --- | --- | --- | --- | --- | --- | --- | --- | --- | --- |
|  |  |  |  | Fe_1_ | Fe_2_ | O_prox_^a^ | O_dist_^a^ | Fe_1_ | Fe_2_ | O_prox_^a^ | O_dist_^a^ |
| Water | 2 | 11 | -4123.567163109 | 1.45 | 1.43 | -0.72 | - | 2.89 | 4.28 | 0.01 | - |
|  | 2 | 9 | -4123.549116201 | 1.45 | 1.33 | -0.72 | - | 2.88 | 2.90 | 0.01 | - |
|  | 2 | 7 | -4123.548490379 | 1.45 | 1.33 | -0.72 | - | 2.88 | 2.91 | 0.01 | - |
|  | 1 | 10 | -4123.899161578 | 1.42 | 1.37 | -0.72 | - | 2.87 | 4.22 | 0.00 | - |
|  | 1 | 8 | -4123.898041297 | 1.43 | 1.21 | -0.72 | - | 2.86 | 4.01 | 0.00 | - |
|  | 0 | 9 | -4124.144849244 | 1.36 | 1.36 | -0.72 | - | 2.88 | 4.22 | 0.00 | - |
|  | 0 | 7 | -4124.130600131 | 1.37 | 1.06 | -0.70 | - | 2.89 | 3.81 | 0.00 | - |
| Hydroxide | 1 | 11 | -4123.274190889 | 1.32 | 1.45 | -0.75 | - | 2.89 | 4.28 | 0.03 | - |
|  | 0 | 10 | -4123.492445712 | 1.34 | 1.36 | -0.75 | - | 2.85 | 4.24 | 0.04 | - |
|  | 0 | 6 | -4123.474574445 | 1.34 | 1.05 | -0.73 | - | 2.87 | 3.80 | -0.01 | - |
| O-O-H | 1 | 11 | -4198.402967073 | 1.45 | 1.18 | -0.36 | -0.19 | 4.27 | 3.86 | -0.20 | -0.58 |
|  | 0 | 10 | -4198.645856136 | 1.36 | 1.44 | -0.44 | -0.43 | 2.85 | 4.27 | 0.03 | 0.05 |
|  | -1 | 9 | -4198.782532053 | 1.34 | 1.41 | -0.48 | -0.43 | 2.87 | 4.27 | 0.03 | 0.05 |
|  | -1 | 5 | -4198.733288905 | 1.37 | 1.07 | -0.49 | -0.43 | 0.95 | 3.80 | 0.07 | 0.02 |
| O-O | 2 | 11 | -4121.005909506 | 1.47 | 1.44 | -0.02 | 0.06 | 4.27 | 4.28 | 0.95 | 1.05 |
|  | 1 | 10 | -4121.335881203 | 1.45 | 1.43 | 0.00 | 0.04 | 4.24 | 4.29 | 0.00 | 0.00 |
|  | 0 | 9 | -4121.578771081 | 1.41 | 1.41 | -0.29 | -0.09 | 4.06 | 4.27 | -0.01 | -0.40 |
| Empty site | 2 | 11 | -3970.682349824 | 1.47 | 1.45 | - | - | 4.27 | 4.28 | - | - |
|  | 0 | 9 | -3971.259761634 | 1.45 | 1.06 | - | - | 4.25 | 3.81 | - | - |
| ^a^ O_prox_ and O_dist_ are named in relation to Fe_1_ in the starting coordinates. | | | | | | | | | | | |
