# Supplementary material for: Structural Characterization of Bacterioferritin from Blastochloris viridis
Source: PLoS One. 2012 Oct 9;7(10):e46992. doi: 10.1371/journal.pone.0046992 (PMC3467274; doi:10.1371/journal.pone.0046992)
Supplement: Table S4 — Structural similarity to the crystallographic positions and key distances in the DFT optimized geometry. (DOC) [file pone.0046992.s007.doc]

**Table S4** Structural similarity to the crystallographic positions and key distances in the DFT optimized geometry.

|  | Charge | Multiplicity | Rmsd (Å) | Fe_1_-ligand distance (Å) | Fe_2_-ligand distance (Å) | | Fe_1_-His54 distance (Å) | Fe_2_-His130 distance (Å) | Fe_1_-Fe_2_ distance (Å) | Remarks |
| --- | --- | --- | --- | --- | --- | --- | --- | --- | --- | --- |
| Water | 2 | 11 | 0.26 | 1.96 | 3.46 | | 1.99 | 2.08 | 3.96 | a |
|  | 2 | 9 | 0.29 | 1.95 | 3.58 | | 1.99 | 1.94 | 4.08 | a |
|  | 2 | 7 | 0.29 | 1.95 | 3.58 | | 1.99 | 1.95 | 4.08 | a |
|  | 1 | 10 | 0.25 | 1.97 | 3.64 | | 1.96 | 1.93 | 3.98 | b |
|  | 1 | 8 | 0.37 | 1.97 | 3.62 | | 1.96 | 2.11 | 3.95 | a |
|  | 0 | 9 | 0.28 | 2.04 | 3.58 | | 1.87 | 1.91 | 3.98 | b |
|  | 0 | 7 | 0.35 | 2.02 | 3.51 | | 1.89 | 2.13 | 3.91 | a |
| Hydroxide | 1 | 11 | 0.33 | 1.78 | 2.74 | | 2.05 | 2.22 | 3.66 | a, c |
|  | 0 | 10 | 0.20 | 1.79 | 3.55 | | 2.03 | 1.93 | 3.93 | b |
|  | 0 | 6 | 0.27 | 1.79 | 3.50 | | 2.05 | 2.17 | 3.89 | a, d |
| O-O-H | 1 | 11 | 0.46 | 2.03 | - | | 2.17 | 2.08 | 3.65 | b, e, f |
|  | 0 | 10 | 0.35 | 1.81 | 2.24 | | 2.00 | 1.99 | 3.71 | b, g |
|  | -1 | 9 | 0.40 | 1.85 | 2.26 | | 1.92 | 1.98 | 3.72 | b, g |
|  | -1 | 5 | 0.40 | 1.82 | 2.23 | | 1.99 | 2.15 | 3.63 | b, g |
| O-O | 2 | 11 | 0.55 | 3.33 | 3.88 | | 2.04 | 2.08 | 4.14 | a, h |
|  | 1 | 10 | 0.50 | 3.22 | 3.83 | | 1.93 | 2.05 | 4.14 | a, h |
|  | 0 | 9 | 0.38 | 1.92 | 3.37 | | 2.17 | 1.96 | 3.95 | a, f |
| Empty | 2 | 11 | 0.33 | - | - | | 2.04 | 2.08 | 4.14 | a |
|  | 0 | 9 | 0.35 | - | - | | 1.93 | 2.16 | 4.10 | a |
| Remarks such as position of Glu94 and unusual position or protonation state of the ligand are indicated: | | | | | | | | | | |
| a Bidentate coordination of Glu94 to Fe2  b Monodentate coordination of Glu94 to Fe2  c Hydroxyl hydrogen orients towards Wat2  d Hydroxyl hydrogen orients towards Glu94 | | | | | | e Proton transfer  f Bridge does not form between Fe1 and Fe2  g Bridge forms between Fe1 and Fe2  h Ligand dissociates | | | | |
