# Supplementary material for: Structural Characterization of Bacterioferritin from Blastochloris viridis
Source: PLoS One. 2012 Oct 9;7(10):e46992. doi: 10.1371/journal.pone.0046992 (PMC3467274; doi:10.1371/journal.pone.0046992)
Supplement: Text S1 — Method description of de novo sequencing by tandem mass spectrometry and DFT calculations. (DOC) [file pone.0046992.s013.doc]

Structural characterization of bacterioferritin from *Blastochloris viridis*

Weixiao Y. Wahlgren^a^, Hadil Omran^a^, David von Stetten^b^, Antoine Royant^b,c^, Sjoerd van der Post^d^ and Gergely Katona^a,*^

^a^ Department of Chemistry and Molecular Biology, University of Gothenburg, Göteborg, Sweden

^b^ European Synchrotron Radiation Facility, Grenoble, France

^c^ Institut de Biologie Structurale Jean-Pierre Ebel, CNRS Commissariat à l'Energie Atomique Université Joseph Fourier, Grenoble, France

^d^ Proteomics Core Facility, University of Gothenburg, Göteborg, Sweden

**Supporting text**

*De novo sequencing by tandem mass spectrometry*

*Bv* Bfr crystals were fished from crystallization mother liquid and moved to fresh drop of water and washed 3 times before they were dissolved in 10 μL of water to a final protein concentration of 1 mg/mL. 0.05 μg purified *Bv* Bfr resolved in 10 µL of 50 mM NH_4_HCO_3_ pH 8.0 was digested overnight at 37 °C with either 5 pg modified trypsin (Promega, Madison, WI) or sequencing grade chymotrypsin (Promega, Madison, WI). The digestion was quenched by the addition of 2 µL 0.1% CF_3_COOH and peptides were extracted using C18 ziptips (Millipore) according to manufacturer’s instructions, lyophilyzed and resolved in 15 µL 0.1% CF_3_COOH. Protein digests were analyzed by nLC-MS/MS (LTQ-Orbitrap XL, Thermo scientific) as described in detail previously with minor modifications; MS/MS spectra were acquired using HCD collision with detection in the Orbitrap mass analyzer at 7500 FWHM resolution. RAW spectral data were converted into peak lists using MSfilereader (version 2.1.0.1131, Thermo scientific) and subjected to *de novo* sequence analysis using PepNovo 3.1 beta with a fragment tolerance of 0.02 Da. Candidate sequences were blasted using the stand-alone version of NCBI-BLAST (version 2.2.22) towards all proteins annotated "bacterioferritin" parsed from NCBInr (9983 entries). Peptide sequences with complete alignment were concatenated into a complete protein sequence using the data from both enzymes to obtain overlap between the different peptides. Spectral data were searched using MASCOT (version 2.2, Matrixscience) to confirm the bacterioferritin sequence for *Blastochloris viridis*. Search parameters were as follows: (i) one missed cleavage trypsin or two for chymotrypsin; (ii) error tolerance 5 p.p.m. (precursor), 0.02 Da (fragment ions); (iii) charge state 2+ , 3+ , 4+ ; and (iv) oxidation methionine (variable) and the ion-score cut-off was set to >50.

*DFT calculations*

Starting coordinates of the DFT simulations were based on the Fe(II) soaked ferroxidase active site as illustrated in Figure S1. Fe_1_ and Fe_2_, the truncated side chains of the coordinating residues were included in all simulated compounds. In compound A-D a water molecule with constrained oxygen position was included close to the putative ligand bound to Fe_1_. The simulated ligand was a water molecule, a hydroxide ion, an O-O and an O-O-H moiety in compounds A-D respectively. Compound E represents a completely empty active site and did not include the ligand and the supporting water molecule. Initial geometries were subjected to unrestricted Hartree-Fock geometry optimizations with different total charge and multiplicity (Table S2 and S3). Most optimization trials were performed on Compound A and B as the electron density of the ligand indicated a single heavy atom, most likely oxygen. As a general observation, highest multiplicity associate with the lowest total energy. Best agreement with the experimental model could be obtained with a hydroxide ion (Compound B, charge=0, multiplicity=10) with an rmsd of 0.20 Å between the atomic coordinates. This can be compared to the estimate of the overall coordinate error of 0.065 Å based on R_free_ value. The presence of a ligand was also required to stabilize the monodentate coordination of Glu94 as all of the tested electronic states of Compound E yielded bidentate coordination. Higher oxidation states of the in Compound A and B at the highest tested multiplicity increased the coordinating distances to Fe_2_, and presumably weaken the coordination, which appear to facilitate Fe(III) release into the cavity via the ferroxidase site.

The solvent environment that stabilizes a bound water or hydroxide ion is not necessarily same as various (hydro-)peroxo compounds (Compound C and D). Therefore the simulation of larger ligands may not yield chemically relevant models. It is worth noting that fully oxidized and mixed valence active sites did not bind oxygen at high multiplicity, but in the fully reduced state the proximal oxygen interacted with Fe_1_, but bridge was not formed. On the other hand bridge formation between Fe_1_ and Fe_2_ was frequently observed in different electronic states of Compound D.

We have not explored the full range of potential charge states and multiplicities and the potential effect of antiferromagnetic coupling. Nevertheless the agreement of experimental model with the DFT optimized coordinates are already close to the experimental error in the most favorable cases. Especially if one considers the difficulty of quantifying the systematic differences between experimental atomic and theoretical nuclear positions. Therefore it would be difficult to identify a better model candidate based on these simple similarity criteria.

**References**

1. Andersch-Bjorkman Y, Thomsson KA, Larssont JMH, Ekerhovd E, Hansson GC (2007) Large scale identification of proteins, mucins, and their O-glycosylation in the endocervical mucus during the menstrual cycle. Molecular & Cellular Proteomics 6: 708-716.

2. Frank A, Pevzner P (2005) PepNovo: De novo peptide sequencing via probabilistic network modeling. Analytical Chemistry 77: 964-973.

3. Murshudov GN, Skubak P, Lebedev AA, Pannu NS, Steiner RA, et al. (2011) REFMAC5 for the refinement of macromolecular crystal structures. Acta Crystallographica Section D-Biological Crystallography 67: 355-367.

4. Ryde U, Nilsson K (2003) Quantum chemistry can locally improve protein crystal structures. Journal of the American Chemical Society 125: 14232-14233.
