# Supplementary material for: Structural Characterization of Bacterioferritin from Blastochloris viridis
Source: PLoS One. 2012 Oct 9;7(10):e46992. doi: 10.1371/journal.pone.0046992 (PMC3467274; doi:10.1371/journal.pone.0046992)
Supplement: Text S2 — Cartesian coordinates derived from the DFT calculations listed in Table S3. (DOC) [file pone.0046992.s014.doc]

**DFT coordinates:**

Water /Charge 2 /Multiplicity 11:

C 6.0 53.0240000000 -8.0290000000 -10.3770000000

C 6.0 53.1524408513 -7.7427864469 -11.8348838385

O 8.0 52.2038769057 -7.8695348944 -12.6583936323

O 8.0 54.2946643815 -7.3935282037 -12.3458132861

H 1.0 49.8140828899 -7.7491107941 -15.2102015933

C 6.0 50.0090000000 -7.0320000000 -16.0070000000

C 6.0 51.4717996453 -6.6524965940 -16.0233739273

O 8.0 51.8314281342 -5.5240696458 -16.5189049148

O 8.0 52.3409932138 -7.4658992709 -15.5686792826

C 6.0 53.5190000000 -10.7090000000 -12.8670000000

C 6.0 54.2156207004 -10.3145697649 -14.1070695658

N 7.0 54.3159638401 -9.0788340448 -14.6362417350

C 6.0 55.0485494035 -9.2523018464 -15.7961846682

N 7.0 55.4344854991 -10.5198336974 -16.0393453881

C 6.0 54.9417438328 -11.2048739267 -15.0206393581

C 6.0 53.1850000000 -0.8760000000 -17.4270000000

C 6.0 53.1703372691 -2.3504008430 -17.2640361657

O 8.0 53.2402902687 -2.9585212065 -16.1424316836

O 8.0 53.1616483945 -3.1189541933 -18.2960265568

C 6.0 57.3660000000 -5.1470000000 -15.3840000000

C 6.0 55.9279747401 -5.5856350913 -15.5974847772

O 8.0 55.2388908247 -4.9745785543 -16.5116416467

O 8.0 55.4375613541 -6.5117626728 -14.9077482554

C 6.0 54.6700000000 -4.0370000000 -20.5040000000

C 6.0 54.1845023049 -5.3879872329 -20.1442144852

N 7.0 53.7083517525 -5.7963973172 -18.9399295553

C 6.0 53.3803975098 -7.1207157874 -19.1296481681

N 7.0 53.6150124226 -7.5993064812 -20.3709129741

C 6.0 54.1080940753 -6.5605635575 -21.0247185375

FE 26.0 53.6265647488 -7.2785702083 -14.1442493265

FE 26.0 53.4831179219 -4.7296823875 -17.1702580359

H 1.0 53.6718369676 -7.3729581026 -9.7935244003

H 1.0 53.3372698677 -9.0607653357 -10.1847974446

H 1.0 49.7610523592 -7.4994260540 -16.9679058696

H 1.0 49.3889630716 -6.1396392172 -15.9067028119

H 1.0 52.5486885115 -10.2168816505 -12.7725429191

H 1.0 54.1315987936 -10.4305206378 -11.9985775229

H 1.0 55.2999511546 -8.4242768584 -16.4413734047

H 1.0 55.0705375442 -12.2747880832 -14.9013442783

H 1.0 52.7794068071 -0.5913454673 -18.3978541580

H 1.0 52.6320572237 -0.3933402763 -16.6184783709

H 1.0 57.3976797066 -4.4879397558 -14.5093705952

H 1.0 57.9858813805 -6.0180986878 -15.1651995161

H 1.0 53.8200041445 -3.3556619983 -20.6315293586

H 1.0 55.3037103005 -3.6056095197 -19.7258212590

H 1.0 52.9694287029 -7.7321057224 -18.3356225297

H 1.0 54.4109989610 -6.6025158003 -22.0649521409

H 1.0 51.9837728738 -7.9220450960 -10.0672848577

H 1.0 53.3879069720 -11.7922971903 -12.8358459158

H 1.0 54.2253519022 -0.5345073813 -17.3651403037

H 1.0 57.7455888622 -4.6039402206 -16.2486878508

H 1.0 55.2222353452 -4.0779381755 -21.4450285772

O 8.0 53.5280000000 -4.4870000000 -11.4590000000

H 1.0 54.4356228271 -4.4020384825 -11.1370254371

H 1.0 53.0137109329 -3.7842270645 -11.0380025862

O 8.0 53.1416843029 -5.4121108280 -13.7940541655

H 1.0 52.4859714916 -4.8946461925 -14.2816286860

H 1.0 53.2959820608 -4.9993613739 -12.8781323084

Water /Charge 2 /Multiplicity 9:

C 6.0 53.0240000000 -8.0290000000 -10.3770000000

C 6.0 53.1458974479 -7.7461505520 -11.8401852312

O 8.0 52.1950864557 -7.8920171861 -12.6564688860

O 8.0 54.2801316669 -7.3801984626 -12.3589156285

H 1.0 49.8329978284 -7.7152815884 -15.1768940853

C 6.0 50.0090000000 -7.0320000000 -16.0070000000

C 6.0 51.4802199636 -6.6339558426 -16.0544082715

O 8.0 51.8425360380 -5.5253060669 -16.5644968005

O 8.0 52.3487958412 -7.4625350319 -15.5947864410

C 6.0 53.5190000000 -10.7090000000 -12.8670000000

C 6.0 54.2151889705 -10.3102345172 -14.1067167753

N 7.0 54.3083906731 -9.0741907623 -14.6378661693

C 6.0 55.0416228521 -9.2458890833 -15.7959959068

N 7.0 55.4355000070 -10.5116274728 -16.0384809835

C 6.0 54.9459692704 -11.1973963956 -15.0191737873

C 6.0 53.1850000000 -0.8760000000 -17.4270000000

C 6.0 53.1544752584 -2.3469427547 -17.2901482276

O 8.0 53.1544250832 -3.0157286023 -16.1942016384

O 8.0 53.2122625941 -3.0956840967 -18.3399587346

C 6.0 57.3660000000 -5.1470000000 -15.3840000000

C 6.0 55.9124693050 -5.5365586462 -15.6325688904

O 8.0 55.2549245407 -4.8621521626 -16.5266707836

O 8.0 55.3972201596 -6.4785160997 -14.9845764565

C 6.0 54.6700000000 -4.0370000000 -20.5040000000

C 6.0 54.1875111658 -5.3709872725 -20.0566146332

N 7.0 53.7489683563 -5.7548678182 -18.8213125258

C 6.0 53.4294060631 -7.0882218841 -18.9517930452

N 7.0 53.6235089275 -7.5952827390 -20.1865572778

C 6.0 54.0846908594 -6.5730354656 -20.8891073747

FE 26.0 53.6015149929 -7.2743325761 -14.1603301439

FE 26.0 53.5191251920 -4.6229800446 -17.2574406402

H 1.0 53.6732178436 -7.3714496381 -9.7969756965

H 1.0 53.3355281240 -9.0607928975 -10.1828657946

H 1.0 49.7560553712 -7.5415407557 -16.9440142475

H 1.0 49.3841352347 -6.1407233138 -15.9319914022

H 1.0 52.5462857637 -10.2202604914 -12.7789385631

H 1.0 54.1250508212 -10.4242358513 -11.9962149697

H 1.0 55.2903257709 -8.4162822020 -16.4392701632

H 1.0 55.0810092541 -12.2661814925 -14.8997240609

H 1.0 52.8154758274 -0.5800891666 -18.4094615426

H 1.0 52.6100601476 -0.3942868155 -16.6327150732

H 1.0 57.4032226339 -4.5176161122 -14.4881709162

H 1.0 57.9537387949 -6.0443901073 -15.1851183260

H 1.0 53.8153116022 -3.3850183724 -20.7246316128

H 1.0 55.2692692844 -3.5358049827 -19.7423117128

H 1.0 53.0609536001 -7.6750269504 -18.1200228058

H 1.0 54.3451451997 -6.6427436760 -21.9391008796

H 1.0 51.9843920335 -7.9205108938 -10.0657342412

H 1.0 53.3933198241 -11.7928412731 -12.8347947149

H 1.0 54.2270633255 -0.5465249387 -17.3272026229

H 1.0 57.7758796976 -4.5881371322 -16.2245733039

H 1.0 55.2546396939 -4.1501169674 -21.4195964309

O 8.0 53.5280000000 -4.4870000000 -11.4590000000

H 1.0 54.4380433770 -4.4608359788 -11.1342680659

H 1.0 53.0452428381 -3.7807887975 -11.0087116157

O 8.0 53.0956144314 -5.4277516585 -13.7928409896

H 1.0 52.5545935736 -4.8648414553 -14.3634577664

H 1.0 53.2471322472 -5.0013069725 -12.8869462296

**Water /Charge 2 /Multiplicity 7:**

C 6.0 53.0240000000 -8.0290000000 -10.3770000000

C 6.0 53.1458852333 -7.7459863681 -11.8401666747

O 8.0 52.1949843388 -7.8914988767 -12.6564263354

O 8.0 54.2801635148 -7.3805630759 -12.3589310202

H 1.0 49.8330068588 -7.7156031776 -15.1771437650

C 6.0 50.0090000000 -7.0320000000 -16.0070000000

C 6.0 51.4798271468 -6.6327936947 -16.0535810799

O 8.0 51.8417476164 -5.5231825674 -16.5618695702

O 8.0 52.3487338191 -7.4617059322 -15.5948961765

C 6.0 53.5190000000 -10.7090000000 -12.8670000000

C 6.0 54.2146813503 -10.3102346948 -14.1069350372

N 7.0 54.3079604539 -9.0742401608 -14.6381215738

C 6.0 55.0411054582 -9.2459174501 -15.7963655594

N 7.0 55.4346601251 -10.5117895728 -16.0388960740

C 6.0 54.9451510467 -11.1975527443 -15.0196170835

C 6.0 53.1850000000 -0.8760000000 -17.4270000000

C 6.0 53.1524152258 -2.3468199011 -17.2886889625

O 8.0 53.1628956096 -3.0138105097 -16.1915211335

O 8.0 53.1986749859 -3.0973729793 -18.3377809945

C 6.0 57.3660000000 -5.1470000000 -15.3840000000

C 6.0 55.9129154054 -5.5376979136 -15.6327473535

O 8.0 55.2559874958 -4.8653060296 -16.5289976773

O 8.0 55.3973102316 -6.4784260823 -14.9834570699

C 6.0 54.6700000000 -4.0370000000 -20.5040000000

C 6.0 54.1886625512 -5.3718044660 -20.0558228826

N 7.0 53.7543033225 -5.7553735841 -18.8239834000

C 6.0 53.4320715824 -7.0923055802 -18.9548275388

N 7.0 53.6242056240 -7.5979914486 -20.1868663260

C 6.0 54.0852120001 -6.5726208170 -20.8896654455

FE 26.0 53.6012375379 -7.2744497579 -14.1602162581

FE 26.0 53.5199955064 -4.6215741811 -17.2551807646

H 1.0 53.6732081615 -7.3715379830 -9.7968559065

H 1.0 53.3355058511 -9.0607971942 -10.1828943099

H 1.0 49.7568568363 -7.5416695021 -16.9441617280

H 1.0 49.3833255534 -6.1412697939 -15.9322341759

H 1.0 52.5464271692 -10.2200327314 -12.7785608173

H 1.0 54.1254948928 -10.4244232066 -11.9964678777

H 1.0 55.2908003719 -8.4161721577 -16.4390835404

H 1.0 55.0801925976 -12.2662339286 -14.9002635124

H 1.0 52.8153926838 -0.5800330758 -18.4093982652

H 1.0 52.6110156490 -0.3930287550 -16.6326902717

H 1.0 57.4024509882 -4.5169567429 -14.4885973029

H 1.0 57.9543325706 -6.0438237609 -15.1842329209

H 1.0 53.8145947690 -3.3871126402 -20.7274092856

H 1.0 55.2671392396 -3.5344789596 -19.7415811902

H 1.0 53.0621570540 -7.6771005880 -18.1223559757

H 1.0 54.3458677979 -6.6420936076 -21.9396252228

H 1.0 51.9843818226 -7.9205239816 -10.0657378135

H 1.0 53.3930502536 -11.7928104292 -12.8347883724

H 1.0 54.2274699364 -0.5478986073 -17.3270059757

H 1.0 57.7759767778 -4.5884642809 -16.2247277274

H 1.0 55.2571084500 -4.1495757916 -21.4181268732

O 8.0 53.5280000000 -4.4870000000 -11.4590000000

H 1.0 54.4383756720 -4.4604922757 -11.1353003670

H 1.0 53.0457177262 -3.7803795520 -11.0086791252

O 8.0 53.0961168956 -5.4273023458 -13.7930170411

H 1.0 52.5513120488 -4.8665611322 -14.3622195318

H 1.0 53.2469518157 -5.0010963009 -12.8868182301

**Water /Charge 1 /Multiplicity 10:**

C 6.0 53.0240000000 -8.0290000000 -10.3770000000

C 6.0 53.1232619252 -7.7258622259 -11.8409511427

O 8.0 52.1676793505 -7.8704341528 -12.6441792110

O 8.0 54.2568577534 -7.3425940985 -12.3415603464

H 1.0 49.8616532939 -7.7144149233 -15.1711354510

C 6.0 50.0090000000 -7.0320000000 -16.0070000000

C 6.0 51.4740088568 -6.6398939924 -16.1086332259

O 8.0 51.8080035358 -5.5835376795 -16.7347568134

O 8.0 52.3605855059 -7.4116380343 -15.6041540692

C 6.0 53.5190000000 -10.7090000000 -12.8670000000

C 6.0 54.1897310028 -10.2745924152 -14.1163323003

N 7.0 54.2586481076 -9.0226445834 -14.6373934228

C 6.0 54.9466611471 -9.1737731572 -15.8264683989

N 7.0 55.3495237028 -10.4250488583 -16.0815253318

C 6.0 54.8912576370 -11.1264108943 -15.0429692954

C 6.0 53.1850000000 -0.8760000000 -17.4270000000

C 6.0 53.1885181911 -2.3219161162 -16.9856194925

O 8.0 53.1506209163 -2.7194837832 -15.8165365411

O 8.0 53.2833231757 -3.1835305841 -17.9857675072

C 6.0 57.3660000000 -5.1470000000 -15.3840000000

C 6.0 55.9112453235 -5.5837471056 -15.5413386367

O 8.0 55.1792999727 -5.0005489775 -16.4242250155

O 8.0 55.4690518223 -6.4946825163 -14.7938996262

C 6.0 54.6700000000 -4.0370000000 -20.5040000000

C 6.0 54.2596484127 -5.4525198182 -20.2602786069

N 7.0 53.8417890896 -5.9376982334 -19.0388060871

C 6.0 53.5552650216 -7.2850513133 -19.2682649529

N 7.0 53.7587175941 -7.6673348283 -20.5073686753

C 6.0 54.2019282562 -6.5351589952 -21.1420571408

FE 26.0 53.6148085043 -7.2335829291 -14.1703728809

FE 26.0 53.5339987977 -4.9390240681 -17.4179494042

H 1.0 53.6703931597 -7.3655546954 -9.8005152873

H 1.0 53.3550622947 -9.0570013465 -10.1986607346

H 1.0 49.7199711607 -7.5317623094 -16.9382704821

H 1.0 49.3949275809 -6.1357367326 -15.9040175898

H 1.0 52.5276434915 -10.2614100638 -12.7640998173

H 1.0 54.1155738542 -10.4121198501 -11.9945654380

H 1.0 55.1365605323 -8.3431107971 -16.4889550793

H 1.0 55.0422134430 -12.1936294285 -14.9384594958

H 1.0 52.6793066947 -0.7698414928 -18.3873163362

H 1.0 52.7147722909 -0.2477430726 -16.6693287168

H 1.0 57.4417680475 -4.4902151515 -14.5113594921

H 1.0 57.9839290949 -6.0264700385 -15.1958908828

H 1.0 53.8289896225 -3.3455188299 -20.4026398727

H 1.0 55.4439496084 -3.7090924186 -19.8013158749

H 1.0 53.1998077652 -7.9337511890 -18.4770383927

H 1.0 54.4654647291 -6.5389557251 -22.1915712897

H 1.0 51.9887307557 -7.9367861056 -10.0469003666

H 1.0 53.4337448134 -11.7970982023 -12.8506514360

H 1.0 54.2239030745 -0.5501608077 -17.5494154922

H 1.0 57.7121972299 -4.6101391175 -16.2666501216

H 1.0 55.0728447511 -3.9445941862 -21.5151970667

O 8.0 53.5280000000 -4.4870000000 -11.4590000000

H 1.0 54.4590413632 -4.5483362238 -11.2059900696

H 1.0 53.1980308951 -3.6477238934 -11.1111976984

O 8.0 53.0447181070 -5.3791864751 -13.8391197543

H 1.0 53.1091633223 -4.6569345696 -14.4908850129

H 1.0 53.2079803448 -4.9969463740 -12.9237248412

**Water /Charge 1 /Multiplicity 8:**

C 6.0 53.0240000000 -8.0290000000 -10.3770000000

C 6.0 53.0912925636 -7.6830642102 -11.8276149993

O 8.0 52.1546282412 -7.9023639231 -12.6321576025

O 8.0 54.1858760904 -7.1774306707 -12.3109426563

H 1.0 49.8411280175 -7.5066006511 -15.0388904496

C 6.0 50.0090000000 -7.0320000000 -16.0070000000

C 6.0 51.4537880527 -6.6010640604 -16.1199345269

O 8.0 51.7611040505 -5.5514623086 -16.7442549727

O 8.0 52.3590766446 -7.3694650099 -15.6154006770

C 6.0 53.5190000000 -10.7090000000 -12.8670000000

C 6.0 54.1324916064 -10.2082186858 -14.1320237612

N 7.0 54.2290378020 -8.9277224222 -14.5920923178

C 6.0 54.8664728886 -9.0385768115 -15.8127189575

N 7.0 55.1937586664 -10.2866285092 -16.1601169512

C 6.0 54.7487195582 -11.0275713403 -15.1370872403

C 6.0 53.1850000000 -0.8760000000 -17.4270000000

C 6.0 53.1856157744 -2.3674021393 -17.2316492859

O 8.0 53.3257636658 -2.9358629650 -16.1095719325

O 8.0 53.1033924919 -3.1056958677 -18.2856421589

C 6.0 57.3660000000 -5.1470000000 -15.3840000000

C 6.0 55.9068897788 -5.5275119127 -15.6004141703

O 8.0 55.3187469496 -5.0925566304 -16.6510433860

O 8.0 55.3392797230 -6.2577081261 -14.7408456623

C 6.0 54.6700000000 -4.0370000000 -20.5040000000

C 6.0 54.1857371404 -5.4177186341 -20.2855419474

N 7.0 53.7353173691 -5.9116633803 -19.1150179026

C 6.0 53.3909949097 -7.2063417240 -19.4005588301

N 7.0 53.5867650194 -7.5897046858 -20.6911666206

C 6.0 54.0747771182 -6.5051344621 -21.2649646262

FE 26.0 53.5458601896 -7.1429061138 -14.1531016401

FE 26.0 53.5188431440 -4.8015264841 -17.3350798088

H 1.0 53.6132326966 -7.3330331351 -9.7775479184

H 1.0 53.4420588071 -9.0303308151 -10.2317104313

H 1.0 49.8070677157 -7.7752651325 -16.7867820335

H 1.0 49.3426386028 -6.1822188236 -16.1543211152

H 1.0 52.5366146338 -10.2620082061 -12.6969951454

H 1.0 54.1554677938 -10.4704910456 -12.0054075131

H 1.0 55.0901710089 -8.1718003737 -16.4163365008

H 1.0 54.8567604806 -12.1049956317 -15.1063250393

H 1.0 52.5469423254 -0.6059837774 -18.2692814386

H 1.0 52.8652271653 -0.3617974931 -16.5196601168

H 1.0 57.4195657876 -4.4211275948 -14.5663908417

H 1.0 57.9328535495 -6.0291550797 -15.0793768842

H 1.0 53.8273610371 -3.3370237049 -20.4828779590

H 1.0 55.3455767670 -3.7246139738 -19.7029105693

H 1.0 53.0012967041 -7.8771415776 -18.6449926862

H 1.0 54.3447346686 -6.4593420744 -22.3135828010

H 1.0 51.9869262032 -8.0367815187 -10.0397693655

H 1.0 53.4196674309 -11.7948812506 -12.9146735490

H 1.0 54.2078873156 -0.5580663109 -17.6578722432

H 1.0 57.7905069812 -4.7069858462 -16.2854027476

H 1.0 55.1824107453 -3.9551356090 -21.4647603340

O 8.0 53.5280000000 -4.4870000000 -11.4590000000

H 1.0 54.2666114002 -5.1001692873 -11.3156004067

H 1.0 53.8571340120 -3.5997129181 -11.2608252991

O 8.0 52.8252839810 -5.3399515723 -13.8245808025

H 1.0 52.8474918959 -4.6498402077 -14.5091998752

H 1.0 53.0391202994 -4.9198983215 -12.9351974056

**Water /Charge 0 /Multiplicity 9:**

C 6.0 53.0240000000 -8.0290000000 -10.3770000000

C 6.0 53.4298252776 -7.6009375485 -11.7532962427

O 8.0 52.6751041969 -7.9072238308 -12.7434478392

O 8.0 54.5047542395 -6.9755376696 -11.9754196070

H 1.0 49.8577080976 -7.7085409461 -15.1668822139

C 6.0 50.0090000000 -7.0320000000 -16.0070000000

C 6.0 51.4821573852 -6.6661210950 -16.1139355072

O 8.0 51.8005887346 -5.6004804203 -16.7670977050

O 8.0 52.3498991976 -7.4173250124 -15.6071219168

C 6.0 53.5190000000 -10.7090000000 -12.8670000000

C 6.0 54.2013115958 -10.2824740900 -14.1307243862

N 7.0 54.4262501518 -8.9776394729 -14.5951064999

C 6.0 55.0979073170 -9.1423160123 -15.7994954765

N 7.0 55.3240570603 -10.3915693620 -16.1130109281

C 6.0 54.7680859622 -11.1075924334 -15.0689160981

C 6.0 53.1850000000 -0.8760000000 -17.4270000000

C 6.0 53.2110093016 -2.3091708216 -16.9135735965

O 8.0 53.1992305599 -2.6237198083 -15.7233828624

O 8.0 53.2840769828 -3.2055706835 -17.8816928251

C 6.0 57.3660000000 -5.1470000000 -15.3840000000

C 6.0 55.9130365956 -5.5878734565 -15.5393946961

O 8.0 55.1649592107 -5.0324411523 -16.4139003575

O 8.0 55.5206513581 -6.5003928177 -14.7628355702

C 6.0 54.6700000000 -4.0370000000 -20.5040000000

C 6.0 54.2697451747 -5.4635489335 -20.2977447650

N 7.0 53.8515169647 -5.9687154430 -19.0670005313

C 6.0 53.5824300551 -7.3168739353 -19.3074938651

N 7.0 53.7960132405 -7.6782372915 -20.5443456779

C 6.0 54.2286557631 -6.5189507470 -21.1738468951

FE 26.0 53.8183556130 -7.2540889769 -14.1823071328

FE 26.0 53.5107351475 -5.0125040306 -17.4527220507

H 1.0 53.5271837498 -7.4210563105 -9.6239920583

H 1.0 53.3139039422 -9.0743138317 -10.2325059396

H 1.0 49.7123277016 -7.5366255008 -16.9327937497

H 1.0 49.4020010002 -6.1307319283 -15.9049290731

H 1.0 52.4962512125 -10.3333409309 -12.7891124255

H 1.0 54.0645939969 -10.3846039628 -11.9723390374

H 1.0 55.3967532193 -8.3005672741 -16.4057144442

H 1.0 54.8118198346 -12.1890617858 -15.0442931372

H 1.0 52.5739225183 -0.8080362138 -18.3286045649

H 1.0 52.8109208643 -0.2024167073 -16.6552229930

H 1.0 57.4402133107 -4.5032826947 -14.5016869126

H 1.0 57.9885747470 -6.0262588272 -15.2131086937

H 1.0 53.8464444855 -3.3414879896 -20.3207447463

H 1.0 55.4948913808 -3.7450053584 -19.8425700653

H 1.0 53.2468168720 -7.9782040065 -18.5184951498

H 1.0 54.4916518670 -6.5182864324 -22.2235801844

H 1.0 51.9407774256 -7.9650885356 -10.2561787343

H 1.0 53.4897571702 -11.8018484612 -12.8454382767

H 1.0 54.2062003430 -0.5809378611 -17.6905302548

H 1.0 57.7024292344 -4.5939680706 -16.2609789290

H 1.0 55.0098319033 -3.9003512260 -21.5342208834

O 8.0 53.5280000000 -4.4870000000 -11.4590000000

H 1.0 54.1517319573 -5.2305260397 -11.3311645021

H 1.0 54.0467172366 -3.6762396124 -11.3815467600

O 8.0 53.1034687405 -5.3610772514 -13.9114132897

H 1.0 53.2233793378 -4.6355421928 -14.5497169235

H 1.0 53.1720324451 -4.9571557375 -12.9990226922

**Water /Charge 0 /Multiplicity 7:**

C 6.0 53.0240000000 -8.0290000000 -10.3770000000

C 6.0 53.3975150931 -7.5911816880 -11.7636246017

O 8.0 52.6306516782 -7.9012692403 -12.7447474121

O 8.0 54.4602565145 -6.9516304978 -11.9942458706

H 1.0 49.9262713446 -7.5860655373 -15.0707782756

C 6.0 50.0090000000 -7.0320000000 -16.0070000000

C 6.0 51.4328258808 -6.5653292140 -16.2226043521

O 8.0 51.6135432746 -5.5302646619 -16.9272619239

O 8.0 52.4004878478 -7.2507139998 -15.7529234760

C 6.0 53.5190000000 -10.7090000000 -12.8670000000

C 6.0 54.1735146089 -10.2371280816 -14.1375076034

N 7.0 54.3641198981 -8.9244932426 -14.6069167709

C 6.0 55.0456012380 -9.0755310257 -15.8008595499

N 7.0 55.3036787416 -10.3218045091 -16.1192422770

C 6.0 54.7616658993 -11.0485106787 -15.0793913182

C 6.0 53.1850000000 -0.8760000000 -17.4270000000

C 6.0 53.1768794158 -2.3741248369 -17.2172999709

O 8.0 53.3447915320 -2.9003516727 -16.0821534292

O 8.0 53.0507869894 -3.0983719217 -18.2675907940

C 6.0 57.3660000000 -5.1470000000 -15.3840000000

C 6.0 55.9125754083 -5.5501991600 -15.6096567108

O 8.0 55.3112454921 -5.0922898700 -16.6221898240

O 8.0 55.4026106008 -6.3416375700 -14.7543390481

C 6.0 54.6700000000 -4.0370000000 -20.5040000000

C 6.0 54.2530087174 -5.4471890944 -20.2914483849

N 7.0 53.8495363452 -5.9671715356 -19.1172491022

C 6.0 53.5957649196 -7.2794315074 -19.3902939139

N 7.0 53.8024312533 -7.6565819653 -20.6885925977

C 6.0 54.2102681656 -6.5414526584 -21.2658355044

FE 26.0 53.7152210780 -7.1921330281 -14.2154837695

FE 26.0 53.4589184439 -4.8647388714 -17.3409549104

H 1.0 53.5032685448 -7.3927769352 -9.6321907560

H 1.0 53.3683664227 -9.0573764449 -10.2304150135

H 1.0 49.7362541255 -7.7066680523 -16.8262810201

H 1.0 49.3223202008 -6.1850748912 -16.0221007518

H 1.0 52.4955258893 -10.3417573203 -12.7619372283

H 1.0 54.0794637871 -10.4023348088 -11.9753650160

H 1.0 55.3373215053 -8.2229889209 -16.3960278299

H 1.0 54.8257974465 -12.1293611375 -15.0535242665

H 1.0 52.6296368876 -0.6157772454 -18.3286034813

H 1.0 52.7725226426 -0.3597191571 -16.5574567897

H 1.0 57.4259194642 -4.5143882581 -14.4931787216

H 1.0 57.9645746921 -6.0406929403 -15.1931345668

H 1.0 53.8650570929 -3.3520076119 -20.2216435460

H 1.0 55.5141391925 -3.7955549163 -19.8466977716

H 1.0 53.2630169296 -7.9695461641 -18.6241829764

H 1.0 54.4694164316 -6.4829406451 -22.3166601682

H 1.0 51.9397872187 -8.0152973397 -10.2496942723

H 1.0 53.4990184633 -11.8018836044 -12.8795253202

H 1.0 54.2218771170 -0.5434682939 -17.5495664466

H 1.0 57.7500376613 -4.6037454385 -16.2467243913

H 1.0 54.9706347766 -3.8725507568 -21.5409159848

O 8.0 53.5280000000 -4.4870000000 -11.4590000000

H 1.0 54.1367527029 -5.2533004346 -11.3897776607

H 1.0 54.0833476753 -3.6973129059 -11.4340439384

O 8.0 52.9429884276 -5.3480056152 -13.8982439208

H 1.0 53.0867857756 -4.6618876693 -14.5740391420

H 1.0 53.0796689155 -4.9291976086 -13.0000521801

**Hydroxide /Charge 1 /Multiplicity 11:**

C 6.0 53.0240000000 -8.0290000000 -10.3770000000

C 6.0 53.4422265699 -7.5987458802 -11.7700806336

O 8.0 52.6389613093 -7.8013066073 -12.7631484446

O 8.0 54.5718700701 -7.1028427664 -12.0193472717

H 1.0 49.8470206941 -7.7841421689 -15.2349421724

C 6.0 50.0090000000 -7.0320000000 -16.0070000000

C 6.0 51.4748010460 -6.7151151890 -16.1275832094

O 8.0 51.7578618052 -5.5914687220 -16.7027311889

O 8.0 52.3658528900 -7.5200446958 -15.7566744214

C 6.0 53.5190000000 -10.7090000000 -12.8670000000

C 6.0 54.2199611903 -10.3528008592 -14.1154006496

N 7.0 54.4372375260 -9.1219591234 -14.6140153009

C 6.0 55.1418665485 -9.3538315645 -15.7766773570

N 7.0 55.3959209104 -10.6545641769 -16.0568273078

C 6.0 54.8382899996 -11.2998521836 -15.0485663304

C 6.0 53.1850000000 -0.8760000000 -17.4270000000

C 6.0 53.1719923561 -2.3427764467 -17.1884845502

O 8.0 53.2547931657 -2.8641934948 -16.0280404035

O 8.0 53.1403428092 -3.1660077724 -18.1681524668

C 6.0 57.3660000000 -5.1470000000 -15.3840000000

C 6.0 55.9609127938 -5.6292629261 -15.6088311442

O 8.0 55.2440699745 -4.9454981998 -16.4170852084

O 8.0 55.5593051075 -6.6685349475 -15.0147989794

C 6.0 54.6700000000 -4.0370000000 -20.5040000000

C 6.0 54.2161276446 -5.3742256951 -20.0332225611

N 7.0 53.8167325339 -5.7468363648 -18.7954693082

C 6.0 53.5040499876 -7.0756406744 -18.9266817649

N 7.0 53.6732185041 -7.6005262200 -20.1707730185

C 6.0 54.1091993641 -6.5747846573 -20.8758078219

FE 26.0 53.8233002104 -7.2046813673 -14.2177874737

FE 26.0 53.4138881824 -4.7129996690 -16.8719919963

H 1.0 53.4706555166 -7.3667633148 -9.6343489971

H 1.0 53.3921996264 -9.0430560080 -10.1917561336

H 1.0 49.6563651650 -7.4295614079 -16.9654308572

H 1.0 49.4435717428 -6.1239165470 -15.7928614672

H 1.0 52.6268066299 -10.0947043137 -12.7303398369

H 1.0 54.1763409077 -10.5302951489 -12.0049445584

H 1.0 55.4764960038 -8.5460483255 -16.4085997935

H 1.0 54.8522944600 -12.3792970084 -14.9494888537

H 1.0 52.7725820883 -0.6435232617 -18.4088733231

H 1.0 52.6325059122 -0.3573874897 -16.6410776454

H 1.0 57.3463409604 -4.4440681749 -14.5428397191

H 1.0 58.0200110169 -5.9766105589 -15.1136871197

H 1.0 53.8034598243 -3.3814634726 -20.6523704173

H 1.0 55.3042536040 -3.5472958430 -19.7616484127

H 1.0 53.1515245424 -7.6630659655 -18.0877855827

H 1.0 54.3505204216 -6.6396558438 -21.9309177441

H 1.0 51.9376335965 -8.0288962734 -10.2827431236

H 1.0 53.2559473591 -11.7687336204 -12.8664344524

H 1.0 54.2239297195 -0.5292764384 -17.3886553283

H 1.0 57.7410252103 -4.6192378552 -16.2610324977

H 1.0 55.2056052223 -4.1332057876 -21.4502779148

O 8.0 53.5280000000 -4.4870000000 -11.4590000000

H 1.0 54.2646816204 -5.1071481722 -11.3441893088

H 1.0 53.8771744521 -3.6143869944 -11.2411961148

O 8.0 53.3199551172 -5.4941923233 -14.2476885387

H 1.0 53.2358804805 -5.0356181884 -13.3829922836

**Hydroxide /Charge 0 /Multiplicity 10:**

C 6.0 53.0240000000 -8.0290000000 -10.3770000000

C 6.0 53.1369895273 -7.8179509804 -11.8697336581

O 8.0 52.1582510566 -7.9715092913 -12.6499092783

O 8.0 54.2777126188 -7.5067840609 -12.3774087668

H 1.0 49.8428390986 -7.5802905839 -15.0789386632

C 6.0 50.0090000000 -7.0320000000 -16.0070000000

C 6.0 51.4828541603 -6.6685554824 -16.1198956202

O 8.0 51.8137887690 -5.5668395912 -16.6742614886

O 8.0 52.3528343127 -7.4893088722 -15.6915369779

C 6.0 53.5190000000 -10.7090000000 -12.8670000000

C 6.0 54.1347771724 -10.3878536096 -14.1725768804

N 7.0 54.2564670428 -9.1712384612 -14.7230750964

C 6.0 54.8759568632 -9.3963401689 -15.9312359887

N 7.0 55.1750961182 -10.6961848674 -16.1901828502

C 6.0 54.7272772784 -11.3328842227 -15.1231327800

C 6.0 53.1850000000 -0.8760000000 -17.4270000000

C 6.0 53.1412534191 -2.3226271516 -17.0034795264

O 8.0 53.0198564866 -2.7244608507 -15.8478145550

O 8.0 53.2759220729 -3.1725586927 -18.0093098454

C 6.0 57.3660000000 -5.1470000000 -15.3840000000

C 6.0 55.9351207014 -5.6368236173 -15.6241807381

O 8.0 55.1904848152 -4.9047083459 -16.3814373849

O 8.0 55.5350684252 -6.6987974181 -15.0886902136

C 6.0 54.6700000000 -4.0370000000 -20.5040000000

C 6.0 54.3243500872 -5.4660947071 -20.2067376156

N 7.0 53.8608391085 -5.9206372465 -18.9691438396

C 6.0 53.6590027277 -7.2828806936 -19.1464561601

N 7.0 53.9515322217 -7.7096442350 -20.3505544291

C 6.0 54.3702536444 -6.5726411691 -21.0210166724

FE 26.0 53.6456308378 -7.2964633878 -14.2328662141

FE 26.0 53.5436728513 -4.9116163473 -17.3603534311

H 1.0 53.1233610384 -7.0450186951 -9.9057705438

H 1.0 53.8465116756 -8.6530260704 -10.0174199818

H 1.0 49.7464116540 -7.6814002667 -16.8491758110

H 1.0 49.3939677594 -6.1331337068 -16.0538059918

H 1.0 52.5501502740 -10.2136505899 -12.7653352081

H 1.0 54.1547494269 -10.3397992817 -12.0533234843

H 1.0 55.1060867746 -8.5891616193 -16.6106349111

H 1.0 54.8045200780 -12.4067814216 -14.9961056043

H 1.0 52.4310853868 -0.6929383528 -18.1976030721

H 1.0 53.0150432352 -0.2208095997 -16.5723101240

H 1.0 57.3389088046 -4.4201191269 -14.5651946687

H 1.0 58.0009309702 -5.9826104664 -15.0901764763

H 1.0 53.8081047464 -3.3737670572 -20.3994557006

H 1.0 55.4527755276 -3.6546909912 -19.8386026814

H 1.0 53.2792673119 -7.9090261144 -18.3482827519

H 1.0 54.6822894313 -6.6146639730 -22.0566799030

H 1.0 52.0599018205 -8.4694499481 -10.1227358329

H 1.0 53.4057749556 -11.7890918130 -12.7528067730

H 1.0 54.1621753324 -0.6616154985 -17.8697108017

H 1.0 57.7521338970 -4.6499776814 -16.2748674716

H 1.0 55.0410588132 -3.9654657304 -21.5300791738

O 8.0 53.5280000000 -4.4870000000 -11.4590000000

H 1.0 53.5313696690 -4.8525145178 -12.3642578089

H 1.0 52.6672372393 -4.0564804895 -11.3949301946

O 8.0 53.2359449293 -5.5928981892 -13.8899738475

H 1.0 52.8498008376 -5.0523589616 -14.5945505563

**Hydroxide /Charge 0 /Multiplicity 6:**

C 6.0 53.0240000000 -8.0290000000 -10.3770000000

C 6.0 53.1027398630 -7.7913343429 -11.8737657403

O 8.0 52.1108323694 -7.9442265818 -12.6304116215

O 8.0 54.2318058019 -7.4458482622 -12.3897520009

H 1.0 49.8751890018 -7.4185881776 -14.9941788191

C 6.0 50.0090000000 -7.0320000000 -16.0070000000

C 6.0 51.4562970211 -6.6293621563 -16.2073952071

O 8.0 51.7002825769 -5.5711429360 -16.8382005747

O 8.0 52.3845573122 -7.4188850962 -15.7878355344

C 6.0 53.5190000000 -10.7090000000 -12.8670000000

C 6.0 54.1434137755 -10.3334855074 -14.1573623265

N 7.0 54.2291243379 -9.1101258320 -14.7001702587

C 6.0 54.9096719176 -9.3008086310 -15.8777442233

N 7.0 55.2769484701 -10.5841050464 -16.1369677657

C 6.0 54.8160318284 -11.2459321625 -15.0902917032

C 6.0 53.1850000000 -0.8760000000 -17.4270000000

C 6.0 53.1796910082 -2.3713198024 -17.2516210463

O 8.0 53.3860150931 -2.9130524807 -16.1344614606

O 8.0 53.0190849493 -3.0905230810 -18.3054393924

C 6.0 57.3660000000 -5.1470000000 -15.3840000000

C 6.0 55.9557775303 -5.6248908032 -15.7173372127

O 8.0 55.3680735841 -5.0482486758 -16.6874846917

O 8.0 55.4433380044 -6.5529933677 -15.0246849845

C 6.0 54.6700000000 -4.0370000000 -20.5040000000

C 6.0 54.1918280180 -5.4261358791 -20.3144019739

N 7.0 53.7858264056 -5.9566192991 -19.1451627364

C 6.0 53.4546457606 -7.2398643090 -19.4531941327

N 7.0 53.6121721652 -7.5942094027 -20.7680900043

C 6.0 54.0680582817 -6.4870706658 -21.3216993863

FE 26.0 53.6026029535 -7.2130535115 -14.2555970618

FE 26.0 53.5061992524 -4.8237345516 -17.3207053529

H 1.0 53.0881298250 -7.0474406133 -9.8957026047

H 1.0 53.8753085412 -8.6229058204 -10.0356468814

H 1.0 49.7668062936 -7.8390189400 -16.7072768631

H 1.0 49.3497614937 -6.1849181167 -16.1927294057

H 1.0 52.5624655472 -10.1964850527 -12.7364441019

H 1.0 54.1678016919 -10.3927482338 -12.0400660470

H 1.0 55.1399435101 -8.4727721885 -16.5311923760

H 1.0 54.9351970432 -12.3163394885 -14.9654861867

H 1.0 52.5530988836 -0.5843037289 -18.2672638719

H 1.0 52.8626163086 -0.3758719293 -16.5119860656

H 1.0 57.2830978583 -4.3915131936 -14.5951008093

H 1.0 57.9665536814 -5.9718936738 -14.9971557409

H 1.0 53.9064037057 -3.3252331752 -20.1698039159

H 1.0 55.5451709955 -3.8496405083 -19.8693975957

H 1.0 53.0958272222 -7.9330616961 -18.7018189038

H 1.0 54.3058754347 -6.4053133733 -22.3762905480

H 1.0 52.0807115748 -8.5095439685 -10.1165274976

H 1.0 53.3900027678 -11.7918288336 -12.8085227460

H 1.0 54.2101822788 -0.5531625652 -17.6415380179

H 1.0 57.8364071408 -4.6919542119 -16.2558173747

H 1.0 54.9380073251 -3.8551019001 -21.5468147397

O 8.0 53.5280000000 -4.4870000000 -11.4590000000

H 1.0 53.4476268886 -4.8984992652 -12.3414907418

H 1.0 52.6983801108 -4.0011203095 -11.3709778491

O 8.0 53.0404277625 -5.5479382571 -13.9268138803

H 1.0 53.2716188075 -4.9167256554 -14.6288717396

**O-O-H /Charge 1 /Multiplicity 11:**

C 6.0 53.0240000000 -8.0290000000 -10.3770000000

C 6.0 53.2472622633 -7.6283989587 -11.8135142810

O 8.0 52.3709261588 -7.8064415577 -12.7219583895

O 8.0 54.3655755819 -7.1351698704 -12.2117256882

H 1.0 49.8475521479 -7.7693180745 -15.2215510540

C 6.0 50.0090000000 -7.0320000000 -16.0070000000

C 6.0 51.4694536479 -6.7864258589 -16.2393775294

O 8.0 51.7680091527 -5.9139078849 -17.1095787219

O 8.0 52.3620996080 -7.4532246797 -15.6112227526

C 6.0 53.5190000000 -10.7090000000 -12.8670000000

C 6.0 54.2135478451 -10.3917372162 -14.1348887855

N 7.0 54.4145709461 -9.1686847544 -14.6560479875

C 6.0 55.1524229931 -9.4020853205 -15.7914799804

N 7.0 55.4284667800 -10.7064777822 -16.0506174901

C 6.0 54.8647208282 -11.3455803131 -15.0422920652

C 6.0 53.1850000000 -0.8760000000 -17.4270000000

C 6.0 53.3632928079 -2.1829811829 -16.7196516944

O 8.0 53.4515994284 -2.0984721648 -15.4254104849

O 8.0 53.4531824548 -3.2431462404 -17.3723104231

C 6.0 57.3660000000 -5.1470000000 -15.3840000000

C 6.0 56.0476453451 -5.8232596572 -15.6439664153

O 8.0 55.4271281284 -5.5004704501 -16.7075209368

O 8.0 55.6022380558 -6.6643432481 -14.8027002476

C 6.0 54.6700000000 -4.0370000000 -20.5040000000

C 6.0 54.2399408604 -5.4475318181 -20.3666411678

N 7.0 53.7684153134 -5.9891555362 -19.2345323555

C 6.0 53.5064595043 -7.3021656466 -19.5675852126

N 7.0 53.7798439278 -7.6378835798 -20.8467543406

C 6.0 54.2383401592 -6.5079298748 -21.3721454685

FE 26.0 53.7403060520 -7.1643255110 -14.1785936229

FE 26.0 53.5960210526 -5.2649679350 -17.2917799632

H 1.0 53.2068095582 -7.1534120764 -9.7472594210

H 1.0 53.7491799468 -8.7973472868 -10.0948256611

H 1.0 49.5455324353 -7.3684750456 -16.9390759070

H 1.0 49.5323005663 -6.0862302172 -15.7346279147

H 1.0 52.6053161404 -10.1211438957 -12.7567845648

H 1.0 54.1751170864 -10.4675784494 -12.0195451239

H 1.0 55.4983817228 -8.5947650174 -16.4211547147

H 1.0 54.8930353078 -12.4231678928 -14.9275708507

H 1.0 52.6900137240 -1.0278118046 -18.3859004599

H 1.0 52.6256840703 -0.1725977072 -16.8087848737

H 1.0 57.1686100371 -4.3038347503 -14.7118877688

H 1.0 58.0597165055 -5.8185893628 -14.8757410756

H 1.0 54.0692388857 -3.3867171816 -19.8638915724

H 1.0 55.7170668528 -3.9294244096 -20.1880349959

H 1.0 53.1061724266 -8.0045009682 -18.8471207968

H 1.0 54.5367752244 -6.4204854654 -22.4099731442

H 1.0 52.0072309085 -8.3901859866 -10.2303569441

H 1.0 53.2885598004 -11.7747969260 -12.8107586926

H 1.0 54.1758141168 -0.4436373817 -17.6090771114

H 1.0 57.7949543661 -4.7655951291 -16.3100216944

H 1.0 54.6062183325 -3.6995059395 -21.5409237426

O 8.0 53.5280000000 -4.4870000000 -11.4590000000

H 1.0 54.2901608839 -5.0538688588 -11.6388422557

H 1.0 53.8947921225 -3.7064176616 -11.0259757846

O 8.0 53.3013032840 -5.2510686554 -14.6864322613

O 8.0 54.0988941651 -4.3166719121 -14.2103779918

H 1.0 53.6470960549 -2.9800350520 -14.9572181632

**O-O-H /Charge 0 /Multiplicity 10:**

C 6.0 53.0240000000 -8.0290000000 -10.3770000000

C 6.0 53.3963256195 -7.7009221200 -11.8053137485

O 8.0 52.5239086416 -7.8127326120 -12.7463855616

O 8.0 54.5550184892 -7.3176623487 -12.1216706847

H 1.0 49.8814274246 -7.8047125151 -15.2495935973

C 6.0 50.0090000000 -7.0320000000 -16.0070000000

C 6.0 51.4796009638 -6.7226308323 -16.2079321558

O 8.0 51.7546161544 -5.6569448052 -16.8661159671

O 8.0 52.3842974056 -7.5050166825 -15.7982993834

C 6.0 53.5190000000 -10.7090000000 -12.8670000000

C 6.0 54.1457462749 -10.3339184705 -14.1604605092

N 7.0 54.3420417419 -9.0829565549 -14.6520215222

C 6.0 54.9661217217 -9.2845598054 -15.8601142922

N 7.0 55.1855607727 -10.5750264191 -16.1788107532

C 6.0 54.6870498493 -11.2415197557 -15.1374125116

C 6.0 53.1850000000 -0.8760000000 -17.4270000000

C 6.0 53.1326047444 -2.2073599920 -16.7221768067

O 8.0 52.7425723690 -2.3206424142 -15.5577212082

O 8.0 53.5452316574 -3.2152938862 -17.4644132599

C 6.0 57.3660000000 -5.1470000000 -15.3840000000

C 6.0 56.0127051467 -5.7798134976 -15.6520907044

O 8.0 55.4377848820 -5.4858705446 -16.7571070194

O 8.0 55.5197626880 -6.5615147195 -14.7933003070

C 6.0 54.6700000000 -4.0370000000 -20.5040000000

C 6.0 54.1569358753 -5.4154584386 -20.2466603092

N 7.0 53.6790869408 -5.8716648290 -19.0339120861

C 6.0 53.3183114926 -7.1822749537 -19.2772498644

N 7.0 53.5258396294 -7.5844544753 -20.5234778034

C 6.0 54.0466818172 -6.4858807426 -21.1387203722

FE 26.0 53.7615846440 -7.2244649474 -14.2001133467

FE 26.0 53.5851731813 -5.0705365227 -17.2103419453

H 1.0 52.7351561479 -7.0894998805 -9.8931001971

H 1.0 53.8888930514 -8.4336900698 -9.8499985360

H 1.0 49.5933859055 -7.3812203435 -16.9579940478

H 1.0 49.4780800060 -6.1208278012 -15.7249891519

H 1.0 52.5396374106 -10.2367514055 -12.7538558059

H 1.0 54.1383826925 -10.3784050777 -12.0241034861

H 1.0 55.2505177204 -8.4635936608 -16.5013855194

H 1.0 54.7058129392 -12.3226997429 -15.0694475980

H 1.0 52.4974655516 -0.8912309279 -18.2784690193

H 1.0 52.9070375438 -0.0731775034 -16.7446801253

H 1.0 57.1893526813 -4.1105680638 -15.0770245833

H 1.0 57.8886780370 -5.6715135611 -14.5848283401

H 1.0 53.9171925728 -3.2751708023 -20.2739243764

H 1.0 55.5517922048 -3.8092764018 -19.8926242508

H 1.0 52.9014282721 -7.8109903591 -18.5013651513

H 1.0 54.3197450901 -6.4929638394 -22.1867198965

H 1.0 52.1797333760 -8.7186557560 -10.3379877120

H 1.0 53.4136872766 -11.7942453599 -12.8053774066

H 1.0 54.1897927334 -0.7074814591 -17.8236253634

H 1.0 57.9591198302 -5.1310457676 -16.3000515357

H 1.0 54.9519619176 -3.9371504607 -21.5548091668

O 8.0 53.5280000000 -4.4870000000 -11.4590000000

H 1.0 53.1538658075 -4.6930759677 -12.3293553863

H 1.0 54.3139313688 -5.0505519572 -11.4462675304

O 8.0 53.0270736525 -5.5755462939 -14.0261494866

O 8.0 53.6068814550 -4.6700536917 -15.0093537768

H 1.0 53.0832462853 -3.8112127966 -14.9433697931

**O-O-H /Charge -1 /Multiplicity 9:**

C 6.0 53.0240000000 -8.0290000000 -10.3770000000

C 6.0 53.4272209715 -7.7053125865 -11.8035468862

O 8.0 52.5764203908 -7.8621235958 -12.7538877467

O 8.0 54.5778726634 -7.2770972833 -12.0709209940

H 1.0 49.9187927636 -7.6749183964 -15.1308223650

C 6.0 50.0090000000 -7.0320000000 -16.0070000000

C 6.0 51.4652799884 -6.6648737127 -16.2291712927

O 8.0 51.6955492652 -5.5734829209 -16.8602657180

O 8.0 52.3920891098 -7.4397782199 -15.8702215620

C 6.0 53.5190000000 -10.7090000000 -12.8670000000

C 6.0 54.1645125688 -10.3505547467 -14.1671692191

N 7.0 54.3547049936 -9.0667893584 -14.6752616340

C 6.0 54.9847444910 -9.2688105239 -15.8837897839

N 7.0 55.2124335456 -10.5327048230 -16.1777283388

C 6.0 54.6994341187 -11.2139343460 -15.0948045492

C 6.0 53.1850000000 -0.8760000000 -17.4270000000

C 6.0 53.0817984059 -2.2210555353 -16.7398825399

O 8.0 52.6306248442 -2.3245719033 -15.5961683534

O 8.0 53.5261929434 -3.2131644398 -17.4700842370

C 6.0 57.3660000000 -5.1470000000 -15.3840000000

C 6.0 56.0090213014 -5.7736219328 -15.6700044959

O 8.0 55.4424897495 -5.4553799517 -16.7740790947

O 8.0 55.5238933756 -6.5547568025 -14.8157297002

C 6.0 54.6700000000 -4.0370000000 -20.5040000000

C 6.0 54.0062605925 -5.3596405922 -20.2902386521

N 7.0 53.5966084435 -5.8513236976 -19.0522099457

C 6.0 53.0665673022 -7.0965846205 -19.3404116572

N 7.0 53.0986111759 -7.4192555101 -20.6150760233

C 6.0 53.6899499570 -6.3240468040 -21.2162810618

FE 26.0 53.7773056969 -7.2847881184 -14.2408513509

FE 26.0 53.5727247085 -5.0943771121 -17.2277106152

H 1.0 52.8413960051 -7.0770986235 -9.8661936136

H 1.0 53.8454223920 -8.5406232184 -9.8700967428

H 1.0 49.6539413624 -7.5813109560 -16.8858232368

H 1.0 49.4049649682 -6.1301211028 -15.8982863138

H 1.0 52.4898695515 -10.3452265818 -12.7960477239

H 1.0 54.0653518162 -10.3084405617 -12.0037538663

H 1.0 55.2468460850 -8.4451129121 -16.5329443232

H 1.0 54.7417608812 -12.2956780371 -15.0345661439

H 1.0 52.6078156166 -0.8908218482 -18.3561330254

H 1.0 52.8180134498 -0.0866030867 -16.7705161135

H 1.0 57.1859954848 -4.1555343056 -14.9540195311

H 1.0 57.9178835391 -5.7478118630 -14.6609435295

H 1.0 54.0687256445 -3.2035600174 -20.1240387117

H 1.0 55.6442513470 -3.9776485389 -20.0005287501

H 1.0 52.6847614894 -7.7405638831 -18.5588142860

H 1.0 53.8617005226 -6.2879368854 -22.2858333554

H 1.0 52.1173461394 -8.6347866646 -10.3469290327

H 1.0 53.5089101362 -11.7992109005 -12.7645865633

H 1.0 54.2283263653 -0.6843410049 -17.6947684160

H 1.0 57.9300831087 -5.0246851026 -16.3103071702

H 1.0 54.8388631379 -3.8784000752 -21.5734067907

O 8.0 53.5280000000 -4.4870000000 -11.4590000000

H 1.0 54.2634502124 -5.1161916136 -11.4256115700

H 1.0 53.1592600745 -4.7019829748 -12.3339043489

O 8.0 53.0404053497 -5.6029257131 -14.0158142671

O 8.0 53.5916535362 -4.6826021042 -15.0119133123

H 1.0 53.0236846330 -3.8664016987 -14.9513636845

**O-O-H /Charge -1 /Multiplicity 5:**

C 6.0 53.0240000000 -8.0290000000 -10.3770000000

C 6.0 53.2708672320 -7.7364726491 -11.8557414868

O 8.0 52.3398172225 -7.7643780799 -12.7206001631

O 8.0 54.4365336645 -7.4566649673 -12.2960316953

H 1.0 49.8706887435 -7.8118813189 -15.2576231184

C 6.0 50.0090000000 -7.0320000000 -16.0070000000

C 6.0 51.4834108536 -6.6844384292 -16.1780795210

O 8.0 51.7285143357 -5.6933757489 -16.9233289732

O 8.0 52.3773852239 -7.4141961974 -15.6420035778

C 6.0 53.5190000000 -10.7090000000 -12.8670000000

C 6.0 54.1223342315 -10.3400664990 -14.1786339129

N 7.0 54.2843372217 -9.0718269507 -14.6566955104

C 6.0 54.8795848208 -9.2444284942 -15.8809441177

N 7.0 55.1109449048 -10.5187988815 -16.2187940423

C 6.0 54.6387894938 -11.2123600706 -15.1565681044

C 6.0 53.1850000000 -0.8760000000 -17.4270000000

C 6.0 53.1470728923 -2.2602889789 -16.7933612400

O 8.0 52.6803771583 -2.3905934183 -15.6419477502

O 8.0 53.6260811079 -3.1904902405 -17.5383035949

C 6.0 57.3660000000 -5.1470000000 -15.3840000000

C 6.0 56.0569645391 -5.8921732593 -15.6478004369

O 8.0 55.5522319925 -5.7525817224 -16.8058565045

O 8.0 55.5493304173 -6.5755029362 -14.7135740655

C 6.0 54.6700000000 -4.0370000000 -20.5040000000

C 6.0 54.1510472497 -5.4241032500 -20.3340703295

N 7.0 53.7513382107 -5.9468433903 -19.1544938638

C 6.0 53.3956162911 -7.2296212899 -19.4586157674

N 7.0 53.5364950230 -7.5777306116 -20.7616208281

C 6.0 54.0075309481 -6.4574390799 -21.3221421431

FE 26.0 53.7302767715 -7.2313234868 -14.1427335690

FE 26.0 53.6704938563 -5.1874085292 -17.1445098668

H 1.0 53.0633600941 -7.0702163163 -9.8494151465

H 1.0 53.8150610904 -8.6721346891 -9.9851959291

H 1.0 49.6061238524 -7.3661145098 -16.9682509335

H 1.0 49.4681636781 -6.1279834477 -15.7165885413

H 1.0 52.5117616008 -10.2951895302 -12.7574828603

H 1.0 54.1195174147 -10.3343948231 -12.0292806691

H 1.0 55.1384552210 -8.4028354852 -16.5073221701

H 1.0 54.6745202075 -12.2951002169 -15.1075659624

H 1.0 52.6383437821 -0.8915822622 -18.3750797026

H 1.0 52.7522534108 -0.1252192873 -16.7634732955

H 1.0 57.0987056405 -4.1420196613 -15.0400478065

H 1.0 57.9487639816 -5.6361985218 -14.6013664982

H 1.0 54.3001435429 -3.3898937262 -19.7047974135

H 1.0 55.7684975875 -4.0265257755 -20.4545442476

H 1.0 53.0249180746 -7.9111833527 -18.7028450258

H 1.0 54.2345368972 -6.3877677673 -22.3807845153

H 1.0 52.0444637413 -8.4895270154 -10.2439718930

H 1.0 53.4622181221 -11.7980988435 -12.7773136026

H 1.0 54.2222412418 -0.6113202728 -17.6557359136

H 1.0 57.9410097937 -5.0576812947 -16.3070365583

H 1.0 54.3879449916 -3.6293236347 -21.4809254272

O 8.0 53.5280000000 -4.4870000000 -11.4590000000

H 1.0 53.6633385152 -4.8551663235 -12.3547981063

H 1.0 52.6448205853 -4.1073221255 -11.5427118169

O 8.0 53.1592755417 -5.5187028469 -13.8964969867

O 8.0 53.6776476983 -4.6707563834 -14.9731044486

H 1.0 53.0990350410 -3.8478533730 -14.9873861592

**O-O /Charge 2 /Multiplicity 11:**

C 6.0 53.0240000000 -8.0290000000 -10.3770000000

C 6.0 53.3971303900 -7.6731866488 -11.7732401856

O 8.0 52.5367770361 -7.2744475717 -12.6420751307

O 8.0 54.5853088411 -7.8080803407 -12.2355616430

H 1.0 49.8117011298 -7.8005591283 -15.2615151648

C 6.0 50.0090000000 -7.0320000000 -16.0070000000

C 6.0 51.4745371675 -6.6224867046 -15.9842241216

O 8.0 51.8117114112 -5.5885955204 -16.6702285033

O 8.0 52.3605442830 -7.2900598476 -15.3637797904

C 6.0 53.5190000000 -10.7090000000 -12.8670000000

C 6.0 54.2793745667 -10.4817596541 -14.1144972889

N 7.0 54.4686506844 -9.2900251779 -14.7232375047

C 6.0 55.2809841404 -9.5727028019 -15.8053509898

N 7.0 55.6111510295 -10.8715388646 -15.9391198966

C 6.0 55.0150749975 -11.4659130999 -14.9161657582

C 6.0 53.1850000000 -0.8760000000 -17.4270000000

C 6.0 53.1853305340 -2.3530920885 -17.3084486391

O 8.0 53.4245029418 -2.9768457967 -16.2148872803

O 8.0 53.0098190931 -3.1168714636 -18.3283531729

C 6.0 57.3660000000 -5.1470000000 -15.3840000000

C 6.0 55.8987558944 -5.5244857016 -15.5628386530

O 8.0 55.2420689698 -5.0591461928 -16.5446297538

O 8.0 55.3446496222 -6.3202063932 -14.7165358559

C 6.0 54.6700000000 -4.0370000000 -20.5040000000

C 6.0 54.1814202190 -5.3882155824 -20.1701759883

N 7.0 53.7167153622 -5.8018860608 -18.9689498254

C 6.0 53.3472499589 -7.1153871735 -19.1763682960

N 7.0 53.5541917808 -7.5784628907 -20.4257184191

C 6.0 54.0641992378 -6.5404329375 -21.0711207261

FE 26.0 53.9030542436 -7.4272972298 -14.1108769828

FE 26.0 53.4255475302 -4.7314677418 -17.2100857309

H 1.0 53.5909216076 -7.4017739035 -9.6805129977

H 1.0 53.3130988579 -9.0639607363 -10.1741477976

H 1.0 49.7688389447 -7.4087941567 -17.0069496074

H 1.0 49.3899353108 -6.1487999307 -15.8326356824

H 1.0 52.6604987387 -10.0402230113 -12.7763771532

H 1.0 54.1833773613 -10.5515552200 -12.0060794312

H 1.0 55.6289662719 -8.8013014960 -16.4804309689

H 1.0 55.0783161652 -12.5322824307 -14.7307867070

H 1.0 52.7451577531 -0.5615043651 -18.3730065655

H 1.0 52.6527088241 -0.4283457227 -16.5839548613

H 1.0 57.4388540196 -4.4531901937 -14.5388664422

H 1.0 57.9504357275 -6.0346681524 -15.1326791822

H 1.0 53.8167762438 -3.3967919446 -20.7681160836

H 1.0 55.1776489895 -3.5584898521 -19.6657442351

H 1.0 52.9226512558 -7.7280853930 -18.3911040551

H 1.0 54.3452648170 -6.5737589166 -22.1175959139

H 1.0 51.9557993732 -7.8922473795 -10.2121051312

H 1.0 53.1671134774 -11.7425751159 -12.8183163451

H 1.0 54.2230655437 -0.5263902334 -17.3745057525

H 1.0 57.7452985344 -4.6654553881 -16.2837274119

H 1.0 55.3372765578 -4.0737017728 -21.3685341684

O 8.0 52.9753830212 -4.3160465098 -13.3793512250

O 8.0 53.3989944730 -3.2528239907 -12.9748500321

**O-O /Charge 1 /Multiplicity 10:**

C 6.0 53.0240000000 -8.0290000000 -10.3770000000

C 6.0 53.3644757702 -7.6577240097 -11.7794821688

O 8.0 52.4666417417 -7.6231883374 -12.6970238773

O 8.0 54.5578820465 -7.4307433244 -12.1682828798

H 1.0 49.8525615506 -7.7793301134 -15.2307368925

C 6.0 50.0090000000 -7.0320000000 -16.0070000000

C 6.0 51.4607281067 -6.5617633413 -15.9994509351

O 8.0 51.7508852500 -5.5097706971 -16.6898304450

O 8.0 52.3650105401 -7.1935047812 -15.3919427402

C 6.0 53.5190000000 -10.7090000000 -12.8670000000

C 6.0 54.2422267883 -10.4743544899 -14.1533697826

N 7.0 54.4765444885 -9.2182802512 -14.6866005185

C 6.0 55.2069195733 -9.4532049318 -15.8469263815

N 7.0 55.4336068880 -10.7269914769 -16.0790542179

C 6.0 54.8410677960 -11.3776979365 -15.0274990263

C 6.0 53.1850000000 -0.8760000000 -17.4270000000

C 6.0 53.1836295575 -2.3558013072 -17.2812351010

O 8.0 53.3882304771 -2.9556001591 -16.1754637469

O 8.0 53.0326322426 -3.1154563913 -18.3060508112

C 6.0 57.3660000000 -5.1470000000 -15.3840000000

C 6.0 55.8848265968 -5.4930424727 -15.5436349037

O 8.0 55.2418893536 -5.0006290585 -16.5326331556

O 8.0 55.3269323500 -6.2573522810 -14.6967780979

C 6.0 54.6700000000 -4.0370000000 -20.5040000000

C 6.0 54.2292034587 -5.4074357504 -20.1330980117

N 7.0 53.7561400698 -5.8015808514 -18.9192225263

C 6.0 53.4792480889 -7.1442256410 -19.0711798289

N 7.0 53.7452451666 -7.6366980103 -20.2922170342

C 6.0 54.2066317025 -6.5831122651 -20.9695758709

FE 26.0 53.8999153982 -7.4726091778 -14.1119445425

FE 26.0 53.4284179598 -4.7446703901 -17.1947400687

H 1.0 53.6391968456 -7.4650911404 -9.6728665335

H 1.0 53.2420470446 -9.0918628006 -10.2315877474

H 1.0 49.7931043664 -7.4654052838 -16.9890905887

H 1.0 49.3485011135 -6.1737238972 -15.8694628062

H 1.0 52.5489930055 -10.2037638943 -12.8432898517

H 1.0 54.1153298955 -10.3591628808 -12.0162276312

H 1.0 55.5575616057 -8.6445931981 -16.4752805444

H 1.0 54.8742754577 -12.4554094980 -14.9374653468

H 1.0 52.7434221242 -0.5854401955 -18.3797224084

H 1.0 52.6468268098 -0.4150777304 -16.5955929750

H 1.0 57.4778743916 -4.5243770695 -14.4909084453

H 1.0 57.9338456354 -6.0658903267 -15.2214356069

H 1.0 53.8086456679 -3.3731285767 -20.6344165114

H 1.0 55.2977949215 -3.5918897093 -19.7272595484

H 1.0 53.0812127611 -7.7464849903 -18.2640465642

H 1.0 54.5143238262 -6.6428896379 -22.0066502168

H 1.0 51.9637159931 -7.8614496615 -10.1863330551

H 1.0 53.3495316812 -11.7787940999 -12.7273295441

H 1.0 54.2210875141 -0.5223425655 -17.3825423653

H 1.0 57.7288846099 -4.6117049081 -16.2595660798

H 1.0 55.2311127553 -4.0680431214 -21.4403260308

O 8.0 52.8550956231 -4.5867156021 -13.1364471386

O 8.0 53.4843219249 -3.5537300319 -13.0249175805

**O-O /Charge 0 /Multiplicity 9:**

C 6.0 53.0240000000 -8.0290000000 -10.3770000000

C 6.0 53.3571674546 -7.7248602967 -11.8078361545

O 8.0 52.4410210582 -7.7853985976 -12.7016106191

O 8.0 54.5378897682 -7.4557255105 -12.1763347245

H 1.0 49.8430599806 -7.7352347352 -15.1916467730

C 6.0 50.0090000000 -7.0320000000 -16.0070000000

C 6.0 51.4720924952 -6.6503724650 -16.0750759591

O 8.0 51.7726607826 -5.5626366539 -16.6825832309

O 8.0 52.3420577081 -7.4271980054 -15.5948903166

C 6.0 53.5190000000 -10.7090000000 -12.8670000000

C 6.0 54.2085403012 -10.4500665913 -14.1551074051

N 7.0 54.3807478480 -9.2454726322 -14.7245173137

C 6.0 55.0836271095 -9.5083914599 -15.8703819093

N 7.0 55.3819154732 -10.8214048228 -16.0809949636

C 6.0 54.8518315854 -11.4263758117 -15.0320028967

C 6.0 53.1850000000 -0.8760000000 -17.4270000000

C 6.0 53.1511266192 -2.3647285562 -17.2753387040

O 8.0 53.4229533236 -2.9449633427 -16.1843842036

O 8.0 52.9177530533 -3.1079696322 -18.2948834860

C 6.0 57.3660000000 -5.1470000000 -15.3840000000

C 6.0 55.9374095189 -5.6107493218 -15.6183655613

O 8.0 55.2279329712 -4.9673768296 -16.4500823258

O 8.0 55.5226171344 -6.6124191067 -14.9587584207

C 6.0 54.6700000000 -4.0370000000 -20.5040000000

C 6.0 54.3354360791 -5.4402125192 -20.1046552037

N 7.0 53.8310838555 -5.8030906135 -18.8636586674

C 6.0 53.6710206084 -7.1780841515 -18.9400230523

N 7.0 54.0270798752 -7.6964201766 -20.0957645379

C 6.0 54.4446696491 -6.6086865797 -20.8327990449

FE 26.0 53.7865590690 -7.2346191866 -14.1662313539

FE 26.0 53.4351378267 -4.7765483785 -17.2441065759

H 1.0 53.7707871094 -7.5961892627 -9.7098949712

H 1.0 53.0157263790 -9.1146683462 -10.2311373243

H 1.0 49.7306718147 -7.5062033472 -16.9546327294

H 1.0 49.3937241483 -6.1390804761 -15.8857555816

H 1.0 52.6164837538 -10.1007784409 -12.7802382936

H 1.0 54.1814334019 -10.4403083100 -12.0336755200

H 1.0 55.3778753246 -8.7235885986 -16.5535285918

H 1.0 54.9067799964 -12.4979933716 -14.8755950619

H 1.0 52.4660786073 -0.5563164804 -18.1838624293

H 1.0 52.9883471807 -0.3856475614 -16.4720713495

H 1.0 57.3608913855 -4.4812590364 -14.5139842160

H 1.0 58.0030363654 -6.0010615312 -15.1506447224

H 1.0 53.7882601724 -3.3927057176 -20.5388601629

H 1.0 55.3806891651 -3.5753804191 -19.8072800185

H 1.0 53.2788612376 -7.7514398499 -18.1094092092

H 1.0 54.8009780413 -6.7208370719 -21.8485964845

H 1.0 52.0287835910 -7.6516833624 -10.1348515061

H 1.0 53.2710053799 -11.7686516161 -12.7702730139

H 1.0 54.1865493740 -0.5915910341 -17.7674991812

H 1.0 57.7449754542 -4.5956622580 -16.2445880759

H 1.0 55.1364574374 -4.0435489093 -21.4933598134

O 8.0 53.3220589154 -5.3868884506 -13.9315241307

O 8.0 52.1570166883 -4.9823960742 -13.6050438719

**Empty /Charge 2 /Multiplicity 11:**

C 6.0 53.0240000000 -8.0290000000 -10.3770000000

C 6.0 53.4273290217 -7.6606374747 -11.7615877725

O 8.0 52.6243921202 -7.1138548970 -12.6069307109

O 8.0 54.5845779559 -7.9342156078 -12.2388224804

H 1.0 49.7814882535 -7.7967432239 -15.2659421654

C 6.0 50.0090000000 -7.0320000000 -16.0070000000

C 6.0 51.4795427255 -6.6415485499 -15.9398522455

O 8.0 51.8466127452 -5.5936978661 -16.5914425365

O 8.0 52.3369698296 -7.3400394713 -15.3150144580

C 6.0 53.5190000000 -10.7090000000 -12.8670000000

C 6.0 54.2682568265 -10.4917069141 -14.1236456057

N 7.0 54.4723792268 -9.2994225804 -14.7345606082

C 6.0 55.2625910838 -9.5981078245 -15.8263494215

N 7.0 55.5672432851 -10.9037774126 -15.9652335639

C 6.0 54.9753480665 -11.4872539778 -14.9355030170

C 6.0 53.1850000000 -0.8760000000 -17.4270000000

C 6.0 53.2117392953 -2.3520330030 -17.3019204555

O 8.0 53.5180214294 -2.9692286130 -16.2200129130

O 8.0 52.9723938125 -3.1235109367 -18.3024738312

C 6.0 57.3660000000 -5.1470000000 -15.3840000000

C 6.0 55.9015759438 -5.5344764766 -15.5405041795

O 8.0 55.2412142525 -5.1186485521 -16.5393045710

O 8.0 55.3519917240 -6.2963789443 -14.6574580534

C 6.0 54.6700000000 -4.0370000000 -20.5040000000

C 6.0 54.1966924179 -5.3920349894 -20.1513853908

N 7.0 53.7221610167 -5.7944220618 -18.9491283812

C 6.0 53.3753686939 -7.1164126137 -19.1387640143

N 7.0 53.6084805108 -7.5985071313 -20.3762610761

C 6.0 54.1124513919 -6.5636777222 -21.0315694612

FE 26.0 53.9291037401 -7.4377826248 -14.0997483035

FE 26.0 53.4292134625 -4.7306968157 -17.1913975956

H 1.0 53.5843098143 -7.4093850073 -9.6668427707

H 1.0 53.3006978423 -9.0659867588 -10.1727021334

H 1.0 49.7971298759 -7.4148123220 -17.0112251671

H 1.0 49.3912832432 -6.1442334697 -15.8540449384

H 1.0 52.6353596337 -10.0693611703 -12.7993290072

H 1.0 54.1795322731 -10.4885601172 -12.0178504566

H 1.0 55.6191780057 -8.8390420216 -16.5109239472

H 1.0 55.0245252465 -12.5537702031 -14.7495317479

H 1.0 52.7807810415 -0.5744799872 -18.3926707233

H 1.0 52.5875977376 -0.4493354756 -16.6155706208

H 1.0 57.4575950571 -4.5012772994 -14.5036921199

H 1.0 57.9661224060 -6.0401183521 -15.1924074229

H 1.0 53.8080931309 -3.3750705731 -20.6608831981

H 1.0 55.2708147969 -3.5904840931 -19.7078997573

H 1.0 52.9439287326 -7.7196059073 -18.3494590626

H 1.0 54.4163336289 -6.6096773749 -22.0708267939

H 1.0 51.9567124186 -7.8717296554 -10.2236260013

H 1.0 53.2061633815 -11.7521936721 -12.7825713599

H 1.0 54.2020779035 -0.4855373219 -17.3099902039

H 1.0 57.7141948702 -4.6209514520 -16.2707358801

H 1.0 55.2525229111 -4.0642297907 -21.4270729226

**Empty /Charge 0 /Multiplicity 9:**

C 6.0 53.0240000000 -8.0290000000 -10.3770000000

C 6.0 53.3571001092 -7.6148424450 -11.7817280183

O 8.0 52.4411324617 -7.5026607064 -12.6705577013

O 8.0 54.5532894582 -7.4342744701 -12.1640323984

H 1.0 49.9056451306 -7.7434961826 -15.1884119885

C 6.0 50.0090000000 -7.0320000000 -16.0070000000

C 6.0 51.4467446184 -6.5344221370 -16.1316276565

O 8.0 51.6840823124 -5.6842796636 -17.0315995666

O 8.0 52.3642056351 -6.9897266767 -15.3678315430

C 6.0 53.5190000000 -10.7090000000 -12.8670000000

C 6.0 54.2351559720 -10.4241202145 -14.1548036636

N 7.0 54.4297205456 -9.1488007144 -14.6841812918

C 6.0 55.1780694522 -9.3572050096 -15.8344294136

N 7.0 55.4555368512 -10.6163933299 -16.0690347921

C 6.0 54.8651306908 -11.2922311862 -15.0148225346

C 6.0 53.1850000000 -0.8760000000 -17.4270000000

C 6.0 53.2272280156 -2.3778184999 -17.3063932496

O 8.0 53.5794288555 -2.9367095863 -16.2301619035

O 8.0 52.9377888877 -3.0857877574 -18.3354010101

C 6.0 57.3660000000 -5.1470000000 -15.3840000000

C 6.0 55.9005933657 -5.5341905816 -15.6032952029

O 8.0 55.3577570665 -5.2312300213 -16.6955536676

O 8.0 55.2987135944 -6.1668441937 -14.6598881436

C 6.0 54.6700000000 -4.0370000000 -20.5040000000

C 6.0 54.2502249566 -5.4459097595 -20.3183358633

N 7.0 53.8483928788 -5.9797508300 -19.1523748511

C 6.0 53.5837587314 -7.2834583789 -19.4502356385

N 7.0 53.7794713582 -7.6417730671 -20.7579362069

C 6.0 54.1926483152 -6.5211118188 -21.3171547202

FE 26.0 53.8510327078 -7.3792529322 -14.1589608587

FE 26.0 53.4841580138 -4.8339789889 -17.3543783981

H 1.0 53.6579400013 -7.4911239886 -9.6686715688

H 1.0 53.2376235482 -9.0967979400 -10.2711924035

H 1.0 49.7161096112 -7.4965998271 -16.9524033755

H 1.0 49.3556302263 -6.1707765959 -15.8461802134

H 1.0 52.5375873592 -10.2253637928 -12.8217930614

H 1.0 54.1051085742 -10.3760878379 -12.0023509982

H 1.0 55.4993870088 -8.5317323621 -16.4580639204

H 1.0 54.9334381041 -12.3695640844 -14.9323815145

H 1.0 52.7604999299 -0.5709517122 -18.3835209255

H 1.0 52.6010414629 -0.4512836211 -16.6058099990

H 1.0 57.4377857409 -4.5313949469 -14.4831130283

H 1.0 57.9560132555 -6.0514739122 -15.2107785208

H 1.0 53.8457787080 -3.3605914356 -20.2521299362

H 1.0 55.4845358509 -3.7898155100 -19.8130577153

H 1.0 53.2495707420 -7.9898713461 -18.7001354589

H 1.0 54.4464328521 -6.4416557666 -22.3682761921

H 1.0 51.9699980033 -7.8542652970 -10.1595816277

H 1.0 53.3683670385 -11.7868261615 -12.7643130449

H 1.0 54.1999733885 -0.4761940358 -17.3331593346

H 1.0 57.7449714000 -4.6031612323 -16.2475814934

H 1.0 55.0084618088 -3.8624074687 -21.5275757422
